# S100A11 protects against neuronal cell apoptosis induced by cerebral ischemia via inhibiting the nuclear translocation of annexin A1

**DOI:** 10.1038/s41419-018-0686-7

**Published:** 2018-05-29

**Authors:** Qian Xia, Xing Li, Huijuan Zhou, Lu Zheng, Jing Shi

**Affiliations:** 1 Department of Neurobiology, Tongji Medical College, Huazhong University of Science and Technology, Wuhan, People’s Republic of China; 2 Key Laboratory of Neurological Diseases, Ministry of Education, Wuhan, People’s Republic of China; 3 Institute for Brain Research, Collaborative Innovation Center for Brain Science, Huazhong University of Science and Technology, Wuhan, People’s Republic of China

## Abstract

The subcellular location of annexin A1 (ANXA1) determines the ultimate fate of neurons after ischemic stroke. ANXA1 nuclear translocation is involved in neuronal apoptosis after cerebral ischemia, and extracellular ANXA1 is also associated with regulation of inflammatory responses. As the factors and mechanism that influence ANXA1 subcellular translocation remain unclear, studies aiming to determine and clarify the role of ANXA1 as a cell fate ‘regulator’ within cells are critically needed. In this study, we found that intracerebroventricular injection of the recombinant adenovirus vector Ad-S100A11 (carrying S100A11) strongly improved cognitive function and induced robust neuroprotective effects after ischemic stroke in vivo. Furthermore, upregulation of S100A11 protected against neuronal apoptosis induced by oxygen-glucose deprivation and reoxygenation (OGD/R) in vitro. Surprisingly, S100A11 overexpression markedly decreased ANXA1 nuclear translocation and subsequently alleviated OGD/R-induced neuronal apoptosis. Notably, S100A11 exerted its neuroprotective effect by directly binding ANXA1. Importantly, S100A11 directly interacted with ANXA1 through the nuclear translocation signal (NTS) of ANXA1, which is essential for ANXA1 to import into the nucleus. Consistent with our previous studies, ANXA1 nuclear translocation after OGD/R promoted p53 transcriptional activity, induced mRNA expression of the pro-apoptotic *Bid* gene, and activated the caspase-3 apoptotic pathway, which was almost completely reversed by S100A11 overexpression. Thus, S100A11 protects against cell apoptosis by inhibiting OGD/R-induced ANXA1 nuclear translocation. This study provides a novel mechanism whereby S100A11 protects against neuronal cells apoptosis, suggesting the potential for a previously unidentified treatment strategy in minimizing apoptosis after ischemic stroke.

## Introduction

Ischemia-reperfusion has long been recognized as a pathological condition that begins with inadequate blood flow to the brain. It then subsequently progresses into a cascade of cellular and molecular events that cause cell death and ultimately lead to many neurological diseases with high morbidity and mortality rates^1–4^. Previous studies have verified that annexin A1 (ANXA1) nuclear translocation induced neuronal apoptosis, particularly cortical, hippocampal, and striatal neurons, after oxygen-glucose deprivation and reoxygenation (OGD/R)^5,6^. This model was applied in the present study to simulate cerebral ischemia in vitro^7–9^. The factors influencing ANXA1 nuclear translocation have rarely been discussed^10^. Therefore, the critical factors and mechanisms underlying ANXA1 nuclear translocation after stroke are being urgently sought.

Structurally, ANXA1 is a well-recognized Ca^2+^-dependent phospholipid-binding protein that is involved in diverse cellular biological events, including cell apoptosis, inflammation, proliferation and differentiation^11–14^. As shown in our recent studies, ANXA1 performs various biological roles, depending on its subcellular localization. According to some researchers, post-translational modification promotes ANXA1 translocation from the cytoplasm to the cell surface, which plays a significant role in anti-inflammatory processes^15,16^. Prednisolone and kirenol promote ANXA1 nuclear translocation, which is associated with attenuating the inflammation induced by collagen-induced arthritis^17^. In DU145 cells, ANXA1 expression is upregulated, leading to cell apoptosis via the mitochondrial pathway^18^. ANXA1 does not contain a classical nucleus localization signal, but our recent study revealed that the amino-acid residue sequence Arg^228^-Phe^237^ (RSFPHLRRVF) of ANXA1 is crucial for the interaction of ANXA1 with importin β and functions as a unique nuclear translocation signal (NTS)^19^. ANXA1 accumulates in the nucleus through the association of this NTS with importin β and subsequently binds to p53, thus increasing p53 transcriptional activity, inducing the pro-apoptotic *Bid* gene expression, and activating the caspase-3 apoptosis pathway, eventually resulting in cell apoptosis after OGD/R^5,6,10^. Therefore, studies aiming to identify the factors that specifically block the nuclear translocation of ANXA1 may provide promising targeted strategies for the treatment of ischemic stroke.

S100A11 is a protein secreted through the non-classical vesicle-mediated pathway that relies on an interaction with Peroxisome biogenesis protein 14, PEX14, a peroxisome membrane protein^20,21^. S100A11 plays a pivotal role in regulating enzyme activity, protein phosphorylation, and calcium homeostasis and interacts with cytoskeletal molecules^22,23^. Several reports indicate that S100A11 also has an essential function in epidermal growth factor (EGF) transport and degradation^24^. Previous studies have shown that S100A11 reduces neuronal death in subjects with Alzheimer’s disease and plays significant roles in disease and the function of the nervous system^25^. As a member of the S100 family of typical EF-hand Ca^2+^-binding proteins, S100A11 interacts with the ANXA1 N-terminal domain through its C-terminal discontinuous domains^26–29^. In particular, Hatoum et al.^30^ found that the interaction between ANXA1 and S100A11 is involved in regulating cell survival by activating p14ARF-p53. However, the effect of S100A11 on cell survival after OGD/R and correlations of S100A11 with OGD/R-induced ANXA1 subcellular transport remain unknown.

In the present study, we investigated the role of S100A11 in the nuclear translocation of ANXA1 and subsequent neuronal apoptosis induced by OGD/R. Interestingly, S100A11 participates in inhibiting the nuclear translocation of ANXA1 and almost completely reversed the increased levels of *Bid* mRNA while suppressing the activation of the caspase-3 apoptosis pathway owing to ANXA1 nuclear translocation, which markedly changed the cell fate after OGD/R and promoted cognitive function in mice after cerebral ischemia-reperfusion injury.

## Results

### S100A11 exerts protective effects on infarct volume, neurological deficit scores, and neuronal survival and neurobehavioral function in mice following focal ischemic injury

**Fig. 1:**
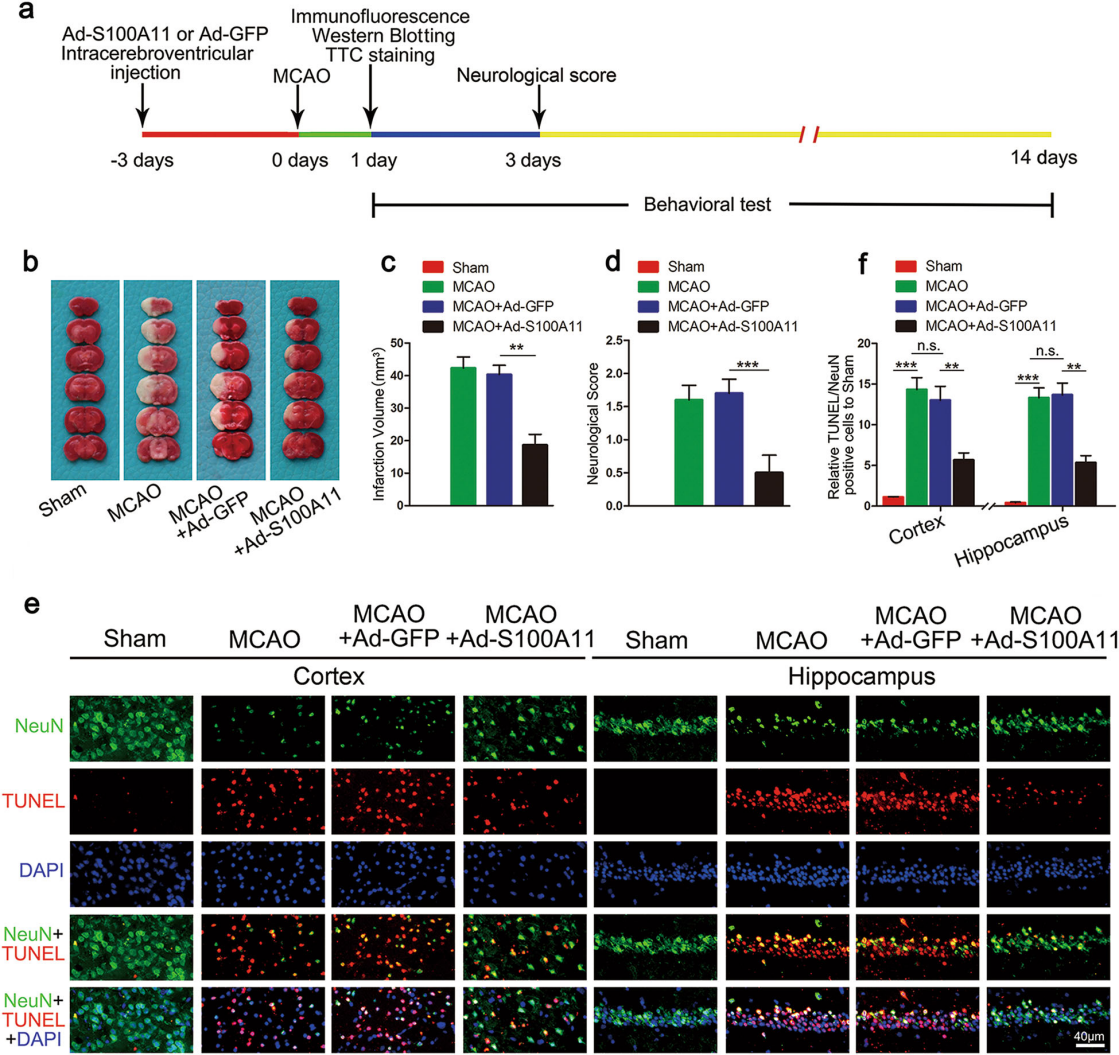
S100A11 significantly reduces infarct volume and cell apoptosis and improves neurological scores in mice after ischemic stroke. **a** Diagram showing the experimental process. **b** Representative images of TTC staining in each treatment group: sham, MCAO, MCAO + Ad-GFP, and MCAO + Ad-S100A11. **c** Statistical analysis of the values shown in **b**; the bar graph shows the infarct volumes in the cortex and the hippocampus 24 h after the 60-min MCAO. *n* = 7 mice; data are presented as the means ± S.E.M. **d** Neurological scores of each group 72 h after reperfusion following MCAO. *n* = 12 mice; data are presented as means ± S.E.M. **e** Representative images of TUNEL staining in the ischemic cortex and the hippocampus. Scale bar = 40 μm. **f** Statistical analysis of representative images of TUNEL staining shown in (**e**). *n* = 6 mice; n.s.: the difference between the two groups was not significant; **P < 0.01; ***P < 0.001

**Fig. 2:**
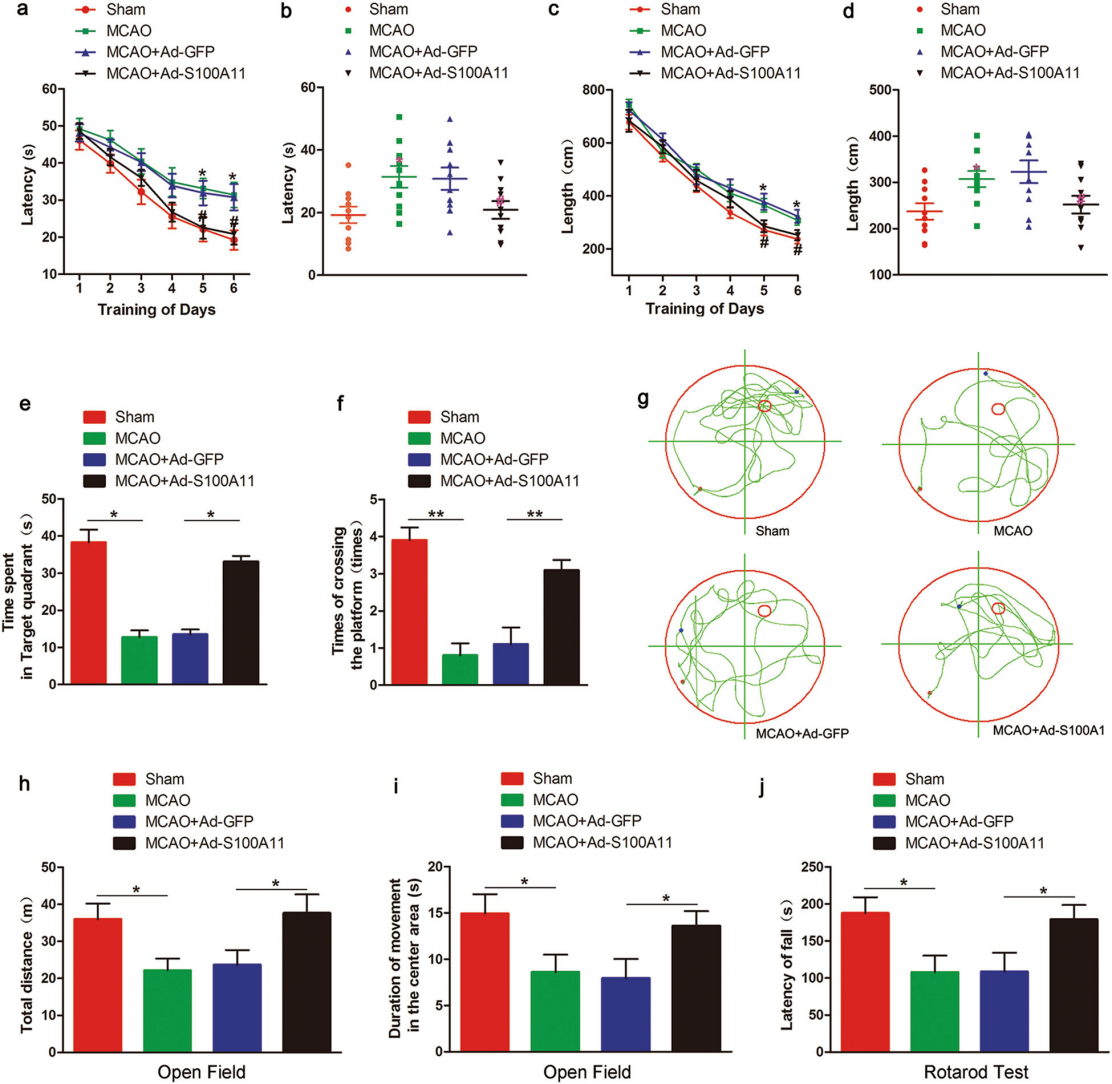
S100A11 enhances learning and memory function and motor ability in mice following ischemic stroke. **a**–**d** Mean escape latency (**a**) and distance swam (**c**) to the hidden platform for the sham, MCAO, MCAO + Ad-GFP, and MCAO + Ad-S100A11 groups over 6 days are plotted against the blocks of trials. Mean escape latency (**b**) and distance swam (**d**) to the hidden platform on day 6 in the Morris water maze test. *P < 0.05 compared with the sham group; ^#^*P* < 0.05 compared with the MCAO group; *n* = 10 mice. **e**, **f** Time (in seconds, s) spent in the target quadrant **(e)**, and the number of times the mouse crossed the target platform location (**f**) during the probe trials on day 7. *n* = 10 mice. **g** Representative path tracings on day 7 during the probe trials. **h**, **i** The total distance traveled (**h**) and the time each mouse spent in the center area (**i**) were analyzed for 10 min during the open field test. *n* = 10 mice. **j** The time mice were able to stay on the rotarod. *n* = 10 mice; data are presented as the means ± S.E.M., *P < 0.05; **P < 0.01

To assess the role of S100A11 in the process of cerebral ischemia, we injected mice with the recombinant adenovirus vector Ad-S100A11 (carrying S100A11) or Ad-GFP 3 days before transient middle cerebral artery occlusion (MCAO) surgery and performed behavioral tests and histological studies at different time points after reperfusion (Fig. 1a). The infection efficiency of Ad-S100A11 in brain tissue was confirmed by western blotting and immunofluorescence staining (Supplementary Figures S1a and 1b). After MCAO, the infarct volume was assessed using 2,3,5-triphenyltetrazolium chloride (TTC) staining (Fig. 1b). Mice pretreated with Ad-S100A11 3 days before ischemia exhibited a marked reduction in the infarct volume (Fig. 1c). Furthermore, animals pretreated with Ad-S100A11 showed better neurological scores after MCAO (Fig. 1d). Similarly, terminal deoxynucleotidyl transferase dUTP nick end labeling (TUNEL)^+^ cells were examined in the cortex and hippocampus after MCAO. A marked reduction in neuron death was observed in Ad-S100A11 group, and Ad-GFP had no effect on neuronal death after MCAO (Fig. 1e, f). Moreover, Ad-S100A11 treatment improved the neurobehavioral function of the mice, including their performance on the Morris water maze test (Fig. 2a–g), the open field test (Fig. 2h, i) and the rotarod test (Fig. 2j), at 7 days post ischemia. Thus, S100A11 overexpression significantly improves neuronal survival, protects against stroke damage, and even improves spatial learning and memory and motor function.

### ANXA1 is involved in the neuroprotection of S100A11 against stroke

**Fig. 3:**
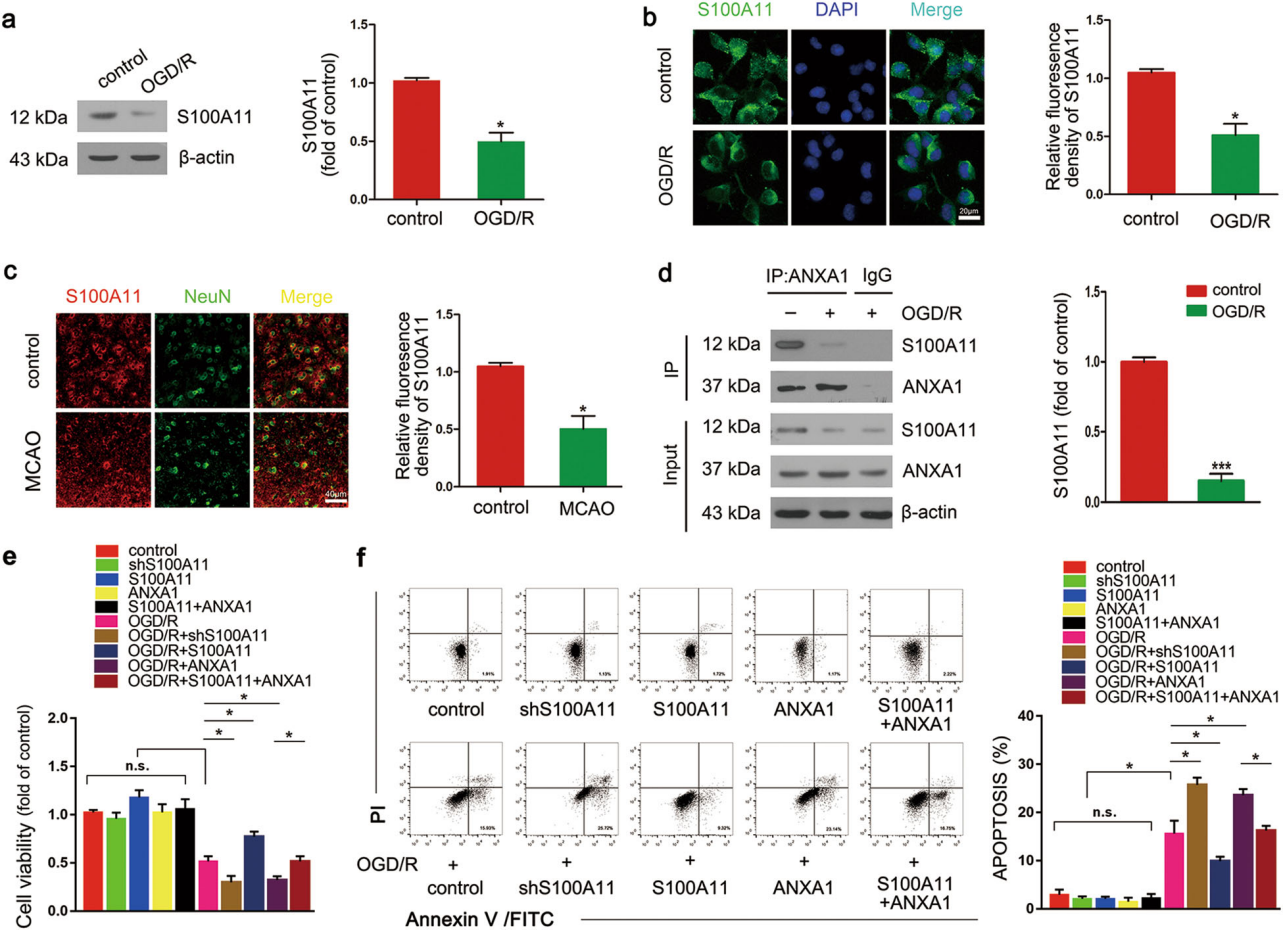
Interaction between S100A11 and ANXA1 decreases and ANXA1 is involved in the neuroprotective effects of S100A11 on stroke in mice. **a**, **b** Western blotting (**a**) and immunofluorescence staining (**b**) indicate the level of S100A11 in N2a cells exposed to OGD for 1 h and reoxygenation for 24 h; quantitative analysis of S100A11 levels is shown. Scale bar = 20 μm. Data are reported as the means ± S.E.M. from three independent experiments. **c** Representative images of S100A11 expression in brain slices from mice subjected to MCAO and quantitative analysis of S100A11 levels are shown. Scale bar = 40 μm. Data are reported as the means ± S.E.M. from three independent experiments. **d** Co-IP showing the interaction of S100A11 with ANXA1 and the quantitative analysis of the binding level. Data are reported as the means ± S.E.M. from three independent experiments. **e**, **f** Representative MTT assays and Annexin V/FITC staining showing the effect of S100A11 and (or) ANXA1 overexpression on N2a cells viability and neuronal apoptosis in cells subjected to OGD/R. **e** N2a cells viability was examined using MTT assays Presented as the means ± S.E.M. from three independent experiments. **f** Cortical neurons apoptosis was presented by Annexin V/FITC assays, cortical neurons were plated in six-well plates as described in Materials and Methods. After 72 h in culture, the cells were transfected with Ad-shS100A11 or Ad-S100A11 and (or) Ad-ANXA1, respectively. After 48 h, the neurons were treated with OGD/R or without OGD/R. Data are reported as the means ± S.E.M. from three independent experiments. n.s.: the difference between the two groups was not significant; *P < 0.05; ***P < 0.001

Western blotting and immunofluorescence staining revealed a 50% decrease in the levels of the endogenous S100A11 protein in N2a cells after OGD/R (Fig. 3a, b). In parallel, immunofluorescence staining showed the same trend in the ischemic brain (Fig. 3c). Annexins and S100 proteins characterize two large but distinct calcium-binding protein families. Moreover, ANXA1 forms heterotetramers with S100A11 (ref. ^24^). For this reason, we proposed that the mechanism by which S100A11 improves neuronal injury may involve ANXA1. We first examined the ANXA1-S100A11 interaction after OGD/R. Co-immunoprecipitation (Co-IP) results showed that the interaction between endogenous ANXA1 and S100A11 was markedly decreased after OGD/R (Fig. 3d), as the levels of ANXA1 and S100A11 when combined exhibited a significantly greater decrease than the level of S100A11 alone. Next, different plasmids encoding short hairpin RNAs targeting S100A11 and ANXA1 were constructed to silence S100A11 or ANXA1 expression and assessed whether the effects of S100A11 on neuronal survival specifically required ANXA1. Western blotting shows cells transfected with shS100A11 #2 and shANXA1 #2 displayed significantly decreased S100A11 and ANXA1 expression, respectively (Supplementary Figures S2a and b), and used in the subsequent experiments. An 3-(4,5-dimethylthiazol-2-yl)-2,5-diphenyltetrazolium bromide (MTT) assay was applied to measure the cells viability after OGD/R. ANXA1 overexpression decreased cell viability after OGD/R, and this ANXA1-induced decrease in cell viability was reversed by S100A11 overexpression (Fig. 3e). Thereafter, we examined neuronal apoptosis using Annexin V/fluorescein isothiocyanate (FITC) assays to investigate the relationship between S100A11 and ANXA1 when protecting against OGD/R-induced injury (Fig. 3f). OGD/R dramatically induced neuronal apoptosis and S100A11 silencing markedly increased the number of apoptotic cells, whereas S100A11 overexpression efficiently decreased the number of apoptotic cells. Overexpression of ANXA1 alone significantly increased cell apoptosis after OGD/R; however, overexpression of both S100A11 and ANXA1 almost completely reversed the increased number of apoptotic cells compared with the overexpression of ANXA1 alone. Based on these results, S100A11 functionally improves cell survival during ANXA1-induced injury after OGD/R.

### S100A11 reduces the expression of apoptosis-related molecules in an ANXA1-dependent manner after OGD/R

**Fig. 4:**
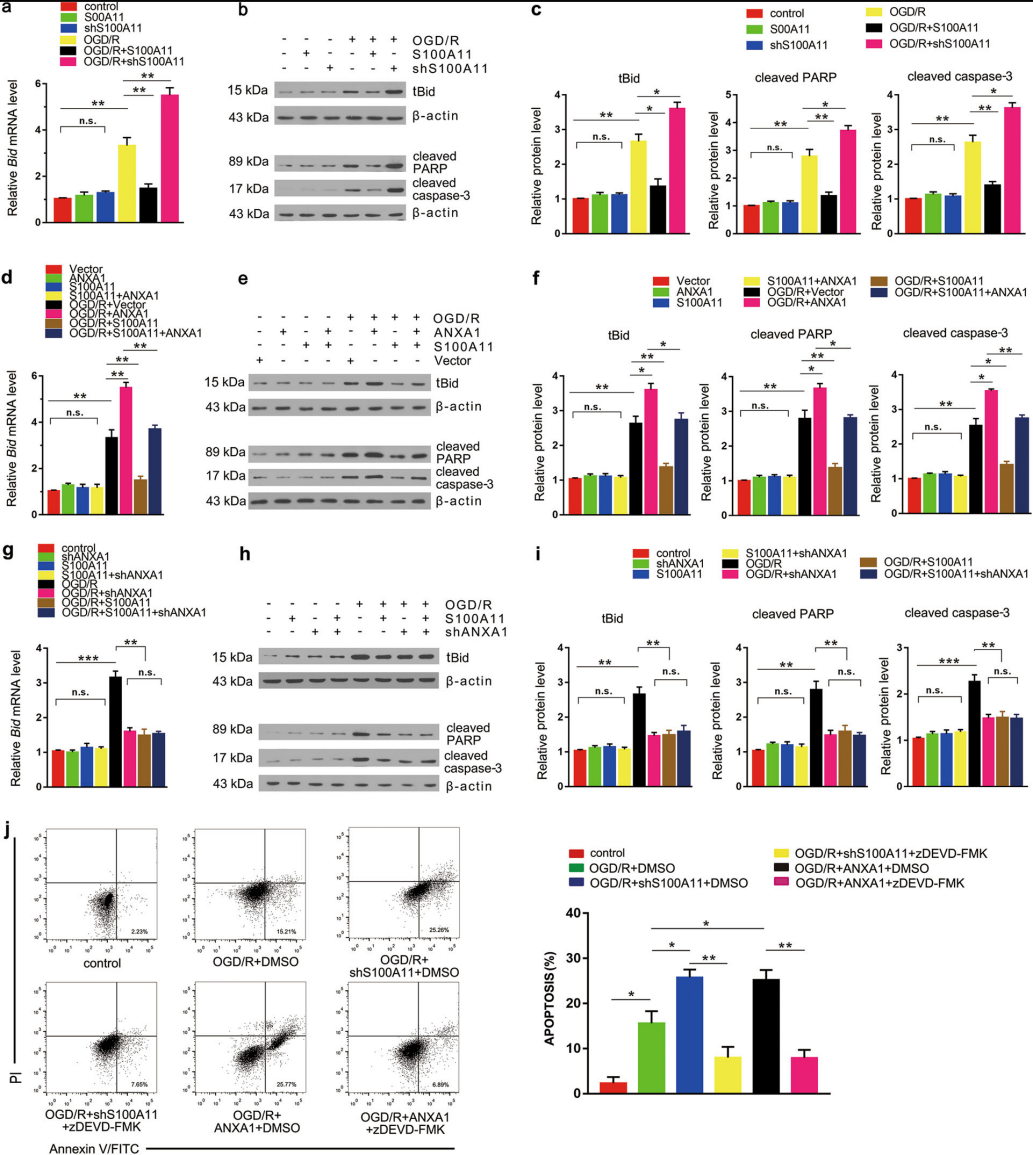
S100A11 overexpression reverses the effect of ANXA1 on increasing OGD/R-induced expression of apoptosis-related proteins and cell apoptosis. **a** qPCR analysis of *Bid* mRNA expression following S100A11 overexpression or silencing in N2a cells treated with or without OGD/R. **b**, **c** Western blotting was performed to evaluate the levels of tBid, cleaved PARP and cleaved caspase-3 protein, followed by statistical analysis of apoptosis-related protein expression. Data are reported as the means ± S.E.M. from three independent experiments. **d**, **g** qPCR analysis of *Bid* mRNA expression in N2a cells transfected with GFP-S100A11 and (or) GFP-ANXA1 plasmids (**d**) and/or shANXA1 (**g**) and treated with or without OGD/R. **e**, **h** Western blots showing the levels of tBid, cleaved PARP and cleaved caspase-3 proteins in N2a cells transfected with GFP-S100A11, GFP-ANXA1 **e** and/or shANXA1 plasmids **h** and treated with or without OGD/R. **f**, **i** Statistical analysis of levels of apoptosis-related proteins shown in (**e**, **h**). **j** Apoptosis was presented by Annexin V/FITC assays in neuronal cells. Cortical neurons apoptosis was presented by Annexin V/FITC assays, cortical neurons were plated in six-well plates as described in Materials and Methods. After 72 h in culture, the cells were transfected with Ad-shS100A11 or Ad-ANXA1, respectively. After 48 h, the neurons were treated with DMSO, the specific caspase-3 inhibitor, zDEVD-FMK (10 μM) with OGD/R or without OGD/R. Data are reported as the means ± S.E.M. from three independent experiments. n.s.: the difference between the two groups was not significant; *P < 0.05; **P < 0.01, and ***P < 0.001

Nuclear translocation of ANXA1 occurs through an interaction with importin β. Then, ANXA1 binds to p53, increases p53 transcriptional activity and induces the expression of the pro-apoptotic protein Bid and activates the caspase-3 apoptosis pathway, eventually resulting in cell apoptosis after OGD/R. N2a cells were exposed to OGD/R to determine whether S100A11 exerted its protective roles by inhibiting ANXA1-induced caspase-3 apoptosis pathway. S100A11 overexpression decreased the expression of the apoptosis-related gene *Bid* compared with the control, as determined using qPCR gene expression analysis. In contrast, S100A11 silencing increased the expression of the *Bid* gene (Fig. 4a). Moreover, western blotting showed that S100A11 overexpression decreased the level of tBid, cleaved PARP and cleaved caspase-3 proteins. In contrast, S100A11 silencing increased the level of tBid, cleaved PARP and cleaved caspase-3 proteins (Fig. 4b, c). Furthermore, based on the results of qPCR gene expression analysis, overexpression of both S100A11 and ANXA1 almost completely reversed the increase in the level of *Bid* mRNA compared with overexpression of ANXA1 alone (Fig. 4d). Moreover, western blotting results indicated that S100A11 overexpression almost completely reversed the increased levels of cell apoptosis-related molecules induced by ANXA1 overexpression after OGD/R (Fig. 4e, f). In addition, the levels of *Bid* mRNA were not different among the S100A11 + shANXA1, S100A11, and shANXA1 groups after OGD/R (Fig. 4g). Similarly, the levels of apoptosis-related proteins showed the same trends as did the mRNAs (Fig. 4h, i). We used Annexin V/FITC assays to confirm that the protection of S100A11 against ANXA1-induced apoptosis after OGD/R specifically required the inhibition of the caspase-3 apoptosis pathway (Fig. 4j). zDVED-FMK, a caspase-3-specific inhibitor, obviously reversed the increased neuronal apoptosis following S100A11 silencing or ANXA1 overexpression after OGD/R. Thus, during the process of OGD/R, ANXA1 increases the expression of apoptosis-related molecules, which is reversed by S100A11 overexpression.

### S100A11 regulates ANXA1 subcellular translocation after OGD/R

**Fig. 5:**
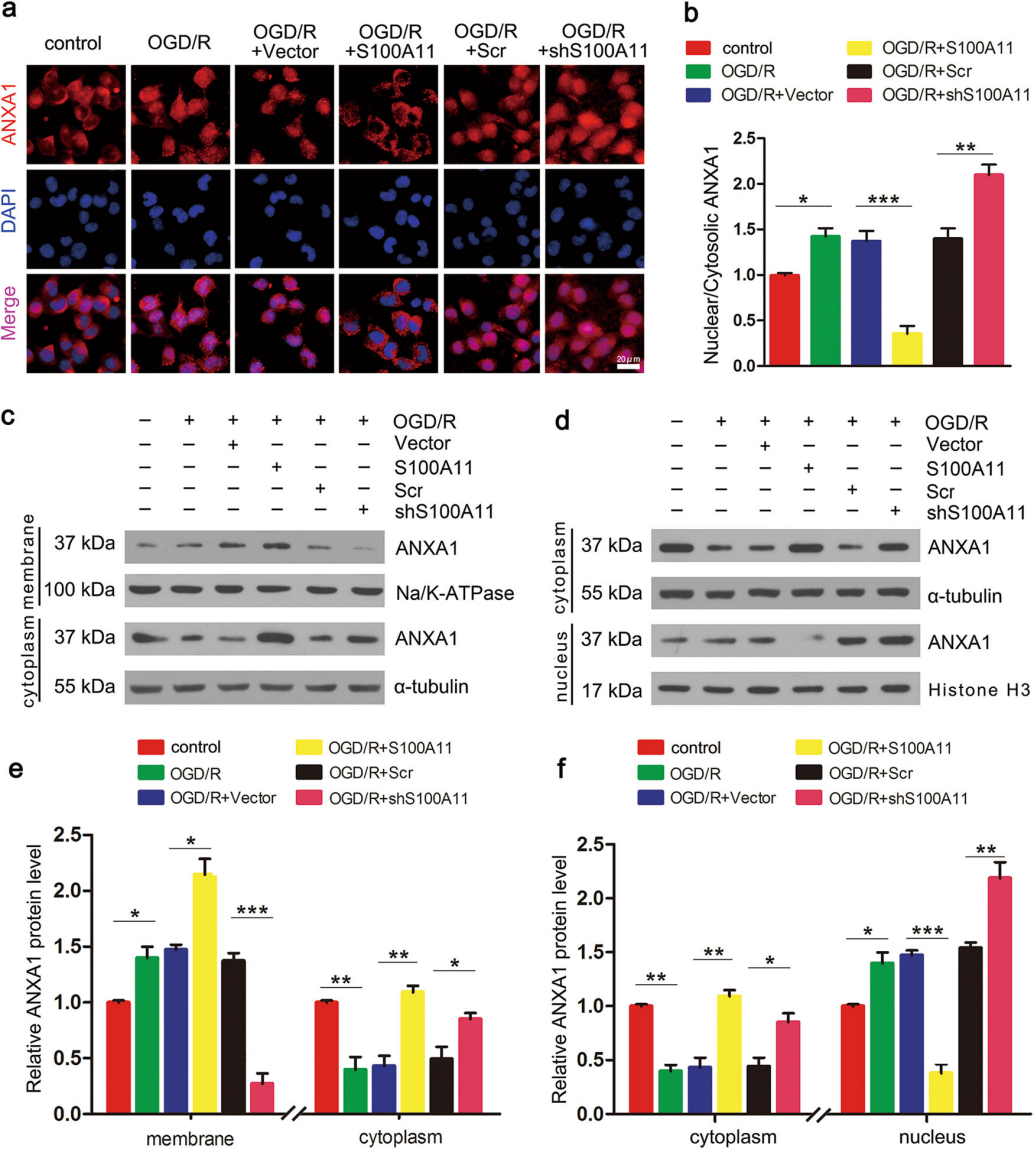
S100A11 overexpression promotes the membrane translocation of ANXA1 and attenuates the nuclear accumulation of ANXA1. **a**, **b** Images of immunofluorescence staining showing the subcellular location of ANXA1 in N2a cells following OGD/R and quantitative analysis of nuclear/cytoplasmic ANXA1 levels. Scale bar = 20 μm. Data are presented as the means ± S.E.M. from three independent experiments. **c**–**f** Western blot showing the subcellular location of ANXA1 in N2a cells transfected with Flag-S100A11 or shS100A11 (**c**, **d**) and subjected to OGD/R, and quantitative analysis of subcellular levels of ANXA1 (**e**, **f**). Scr: scrambled shRNA; *P < 0.05; **P *<* 0.01, and ***P *<* 0.001; data are reported as the means ± S.E.M. from three independent experiments

Based on the aforementioned results, ANXA1 performs its biological roles dependent on its subcellular localization. In addition, more of ANXA1 protein has been observed in the cytosolic fraction under normal conditions in previous studies^31^. N2a cells were first subjected to OGD/R and transfected with or without shS100A11 or S100A11 to examine the effect of S100A11 on ANXA1 subcellular transport. Both immunofluorescence staining and western blotting revealed ANXA1 nuclear accumulation; however, a small amount of ANXA1 was present in the cytoplasm (Fig. 5a–f). Immunofluorescence staining indicated that ANXA1 was predominantly located in the cytoplasm after S100A11 overexpression. Most of the ANXA1 protein had primarily accumulated in the nucleus, but only a small amount of the ANXA1 protein was detected in the cytoplasm after S100A11 silencing following OGD/R (Fig. 5a, b). Similar findings were also obtained using western blotting (Fig. 5c–f). The accumulation of ANXA1 in the cell membrane and nucleus increased, but markedly reduced in the cytoplasm. However, after overexpressing S100A11, ANXA1 was primarily located in the cell membrane and cytoplasm, whereas its levels in the cell nucleus decreased. In contrast, upon S100A11 silencing, ANXA1 was primarily detected in the nuclear fraction and was detected at lower levels in the cell membrane fraction. Based on these results, S100A11 regulates ANXA1 subcellular translocation after OGD/R.

### S100A11 promotes ANXA1 membrane translocation through a mechanism involving PEX14 and inhibits the nuclear translocation of ANXA1

**Fig. 6:**
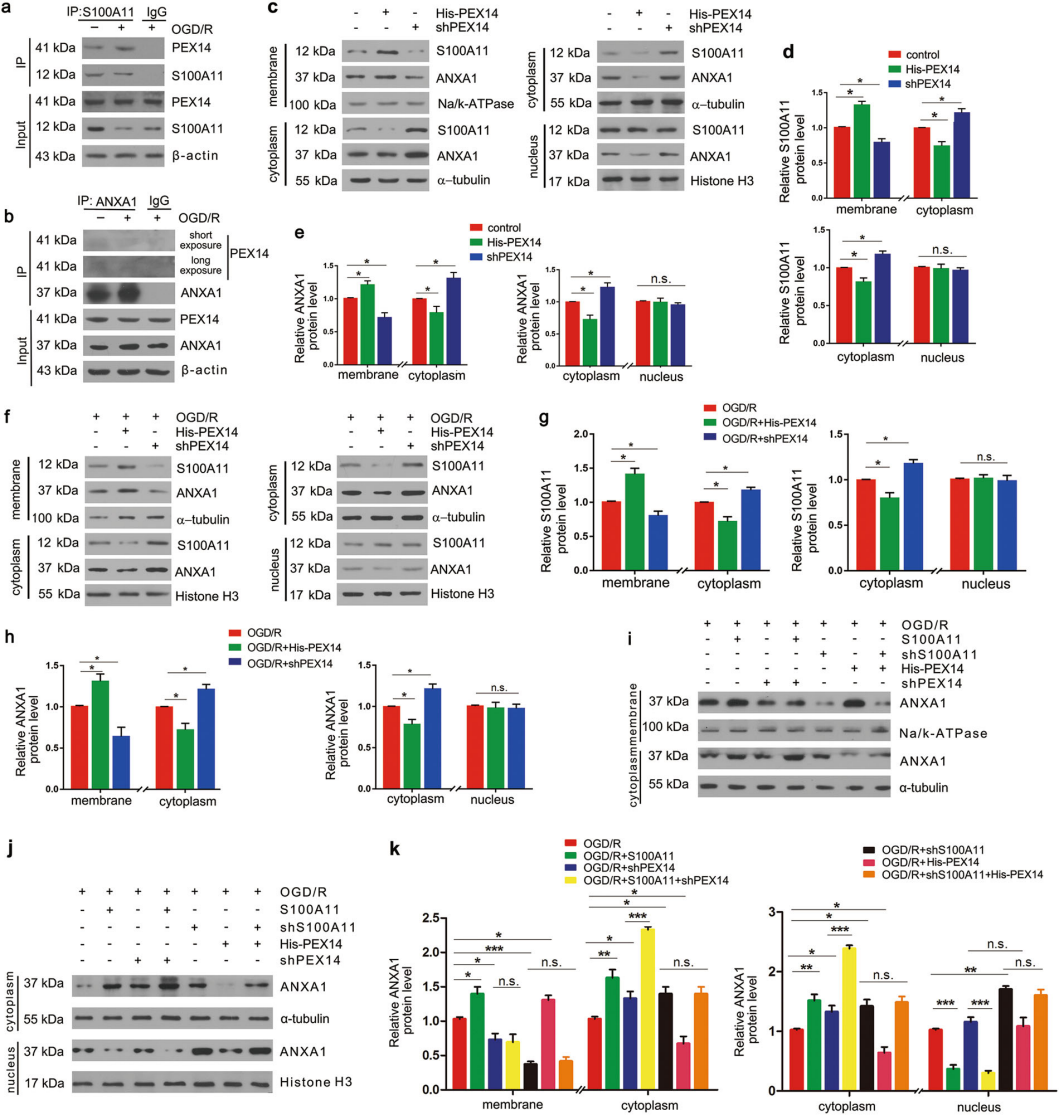
S100A11 enhances the membrane translocation of ANXA1 through a mechanism involving PEX14 and actively inhibits the nuclear translocation of ANXA1. **a**, **b** Co-IP showing the binding of S100A11 (**a**) or ANXA1 (**b**) to PEX14. **c**–**e** Western blot (**c**) showing the subcellular location of S100A11 and ANXA1 in N2a cells treated with His-PEX14 or shPEX14 and the quantitative analysis of subcellular levels of S100A11 (**d**) and ANXA1 (**e**). **f**–**h** Western blot (**f**) showing the subcellular localization of S100A11 and ANXA1 in N2a cells transfected with His-PEX14 or shPEX14 after OGD/R and the quantitative analysis of subcellular levels of S100A11 (**g**) and ANXA1 (**h**). Data are reported as the means ± S.E.M. from three independent experiments. **i**–**k** Western blot showing the subcellular localization of ANXA1 in N2a cells transfected with shS100A11 or S100A11 and/or PEX14 or shPEX14 after OGD/R, and quantitative analysis of subcellular levels of ANXA1. *P < 0.05; **P < 0.01; ***P < 0.001; n.s.: the difference between the two groups was not significant. Data are reported as the means ± S.E.M. from three independent experiments

Next, we sought to determine the reason why the nuclear ANXA1 level was reduced by S100A11 overexpression. One possibility is that the increase in ANXA1 membrane and cytoplasm translocation caused by S100A11 overexpression leads to its reduction in nuclear fractions due to passive diffusion based on molecular concentrations^32^. Another possibility is that S100A11 directly participates in some part of the process of inhibiting ANXA1 nuclear translocation. We examined the localization of ANXA1 in cytoplasmic and nuclear fractions when S100A11 membrane translocation was inhibited and observed a reduction in ANXA1 membrane translocation (Fig. 6). PEX14, Peroxisome biogenesis protein 14, functions as an essential vesicle protein for the membrane translocation and secretion of S100A11^20^. We examined interactions among endogenous S100A11, ANXA1, and PEX14 using Co-IP and found that the interaction between S100A11 and PEX14 increased following OGD/R (Fig. 6a). On the other hand, ANXA1 and PEX14 did not form a direct complex in N2a cells after OGD/R or not (Fig. 6b). Next, we evaluated the silencing efficiency of shPEX14 using real-time PCR analysis and the level of PEX14 after PEX14 overexpression by western blotting to confirm the role of PEX14 in inducing S100A11 membrane translocation (Supplementary Figure S3). The application of shPEX14 #2 efficiently decreased the *PEX14* mRNA level, and His-PEX14 significantly increased PEX14 expression in vitro. Subsequently, we transfected His-PEX14 or shPEX14 into N2a cells, and western blotting analysis (Fig. 6c–e) showed that PEX14 overexpression increased S100A11 and ANXA1 membrane accumulation and decreased their levels in the cytoplasm. In contrast, PEX14 silencing decreased S100A11 and ANXA1 membrane accumulation and increased their levels in the cytoplasm. Furthermore, the same phenomena were observed under OGD/R conditions (Fig. 6f–h). However, interestingly, nuclear S100A11 and ANXA1 levels did not change under any condition. Therefore, PEX14 directly promoted S100A11 membrane translocation but had no effect on S100A11 and ANXA1 nuclear translocation. Meanwhile, we postulated that S100A11 increased ANXA1 membrane accumulation involved in PEX14. We next examined ANXA1 subcellular translocation when increasing cytoplasmic accumulation of S100A11 after shPEX14 to clarify the role of S100A11 in regulating the subcellular translocation of ANXA1 after OGD/R (Fig. 6i–k). The trend of ANXA1 translocation after S100A11 overexpression or silencing was consistent with the data shown in Fig. 5c–f. Moreover, significant differences in ANXA1 levels in any cell fraction were not observed between the OGD/R + shS100A11 and OGD/R + shS100A11 + PEX14 groups, indicating that S100A11, but not PEX14, played a leading role in regulating ANXA1 translocation. Furthermore, the increase in ANXA1 membrane translocation induced by S100A11 overexpression was reversed by shPEX14, leading to the accumulation of ANXA1 in the cytoplasm. However, a change in ANXA1 levels in the nucleus was not observed after OGD/R, implying that the role of S100A11, which promotes ANXA1 membrane migration, depends on PEX14, and directly inhibits ANXA1 nuclear translocation.

### A direct interaction between S100A11 and ANXA1 inhibits the nuclear translocation of ANXA1

**Fig. 7:**
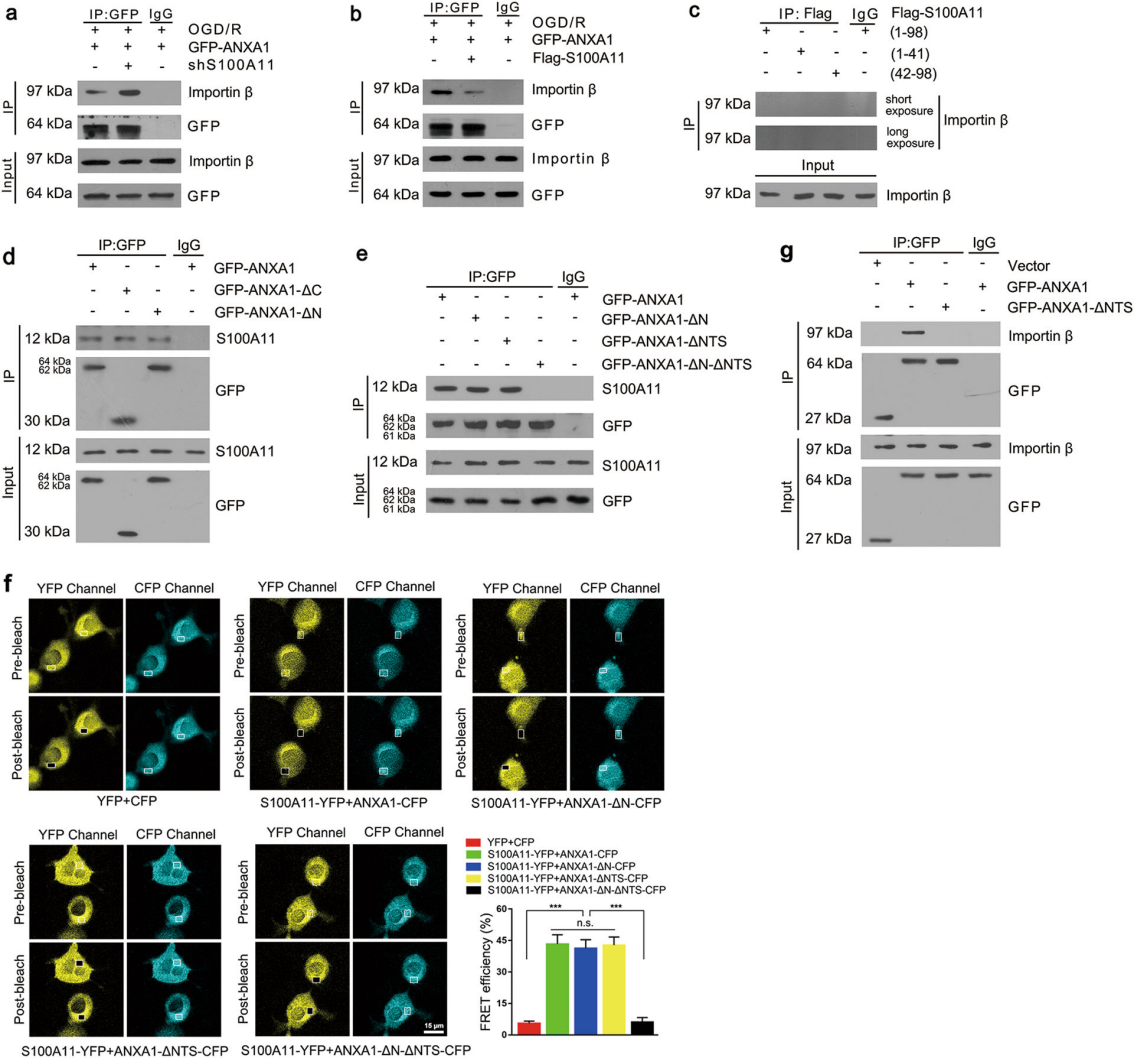
S100A11 blocks the ANXA1-importin β interaction by competing for the NTS sequence of ANXA1. **a**, **b** Co-IP showing the interaction of ANXA1 with importin β in N2a cells transfected with GFP-ANXA1 and shS100A11 (**a**) and/or Flag-S100A11 (**b**) plasmids after OGD/R. **c** Co-IP showing the interaction of S100A11 with importin β in N2a cells transfected with Flag-S100A11 (WT), (1-41), and (42–98) plasmids. **d** Co-IP showing the interaction of S100A11 with ANXA1 in N2a cells transfected with GFP-ANXA1 (WT), (-ΔN), and (-ΔC) plasmids. **e** Co-IP showing the interaction of S100A11 with ANXA1 in N2a cells transfected with GFP-ANXA1 (WT), (-ΔN), (-ΔNTS), and (-ΔN-ΔNTS) plasmids. **f** N2a cells coexpressing S100A11-YFP and CFP-tagged mutant constructs of ANXA1 were serum deprived, fixed, and examined by confocal microscopy. Representative images depicting an increase in donor intensity (CFP) after bleach of acceptor (YFP) within the ROI (white boxes) in N2a cells and bar graph summary of the FRET efficiency. Note that donor and acceptor intensities did not change significantly in areas not subjected to photobleaching (outside the white box). Values shown are mean ± S.E.M; ***P *<* 0.001, Scale bar = 15 μm. **g** Co-IP showing the interaction of importin β with ANXA1 in N2a cells transfected with GFP-ANXA1 (WT), (−ΔNTS) plasmids

Based on our results, nuclear translocation of ANXA1 does not depend on a classic nuclear-migration pathway^6^ but rather depends on the interaction with importin β through the NTS of ANXA1. We applied Co-IP analyses to evaluate whether S100A11 directly regulates the association of importin β with ANXA1 and determine the mechanism by which S100A11 inhibits the nuclear translocation of ANXA1. The interaction of ANXA1 with importin β was markedly increased after S100A11 silencing, but significantly decreased after S100A11 overexpression after OGD/R (Fig. 7a, b). These novel findings provide insight demonstrating that S100A11 may inhibit the nuclear translocation of ANXA1 by interfering with the specific domain of ANXA1 that binds to importin β.

According to previous reports, S100A11-(42–98) contains an ANXA1-binding domain^33^. Next, mutations were generated in two regions of the protein: regions from 1 to 41 and 42 to 98 amino acids. None of S100A11 (wild-type (WT), 1–41, and 42–98) interacted with importin β (Fig. 7c). We also transfected different constructs of green fluorescent protein (GFP)-tagged mutant ANXA1 (GFP-ANXA1, GFP-ANXA1-ΔN, and GFP-ANXA1-ΔC) into N2a cells. All three different ANXA1 constructs interacted with S100A11, suggesting that the N-terminal and C-terminal domains of ANXA1 were accessible to S100A11 for the interaction (Fig. 7d). Surprisingly, the ANXA1 N-terminal domain is a known functional binding domain of S100A11. Moreover, according to our previous study, the ANXA1 C-terminal domain contains the NTS sequence of ANXA1. Thus, S100A11 may inhibit the nuclear translocation of ANXA1 by competing with importin β. Based on this possibility, we generated different plasmids containing GFP-tagged WT, GFP-ANXA1-ΔN, GFP-ANXA1-ΔNTS, and GFP-ANXA1-ΔN-ΔNTS and transfected the constructs into N2a cells. Interestingly, the result confirmed our hypothesis (Fig. 7e). All the ANXA1 mutants bound to S100A11, with the exception of the GFP-ANXA1-ΔN-ΔNTS mutant, suggesting that both the N-terminal domain and NTS of ANXA1 interact with S100A11. The results were further confirmed by Fluorescence resonance energy transfer (FRET) assays (Fig. 7f) and Bimolecular Fluorescence Complementation assay (Supplementary Figure 4). Furthermore, GFP-ANXA1, but not the GFP-ANXA1-ΔNTS mutant, bound to importin β (Fig. 7g). Therefore, the enhanced ANXA1-S100A11 interaction restrained ANXA1 translocation from the cytoplasm to the nucleus by competing with importin β for the NTS domain of ANXA1.

### S100A11-(42–98) inhibits ANXA1 nuclear translocation and protects against cell apoptosis

**Fig. 8:**
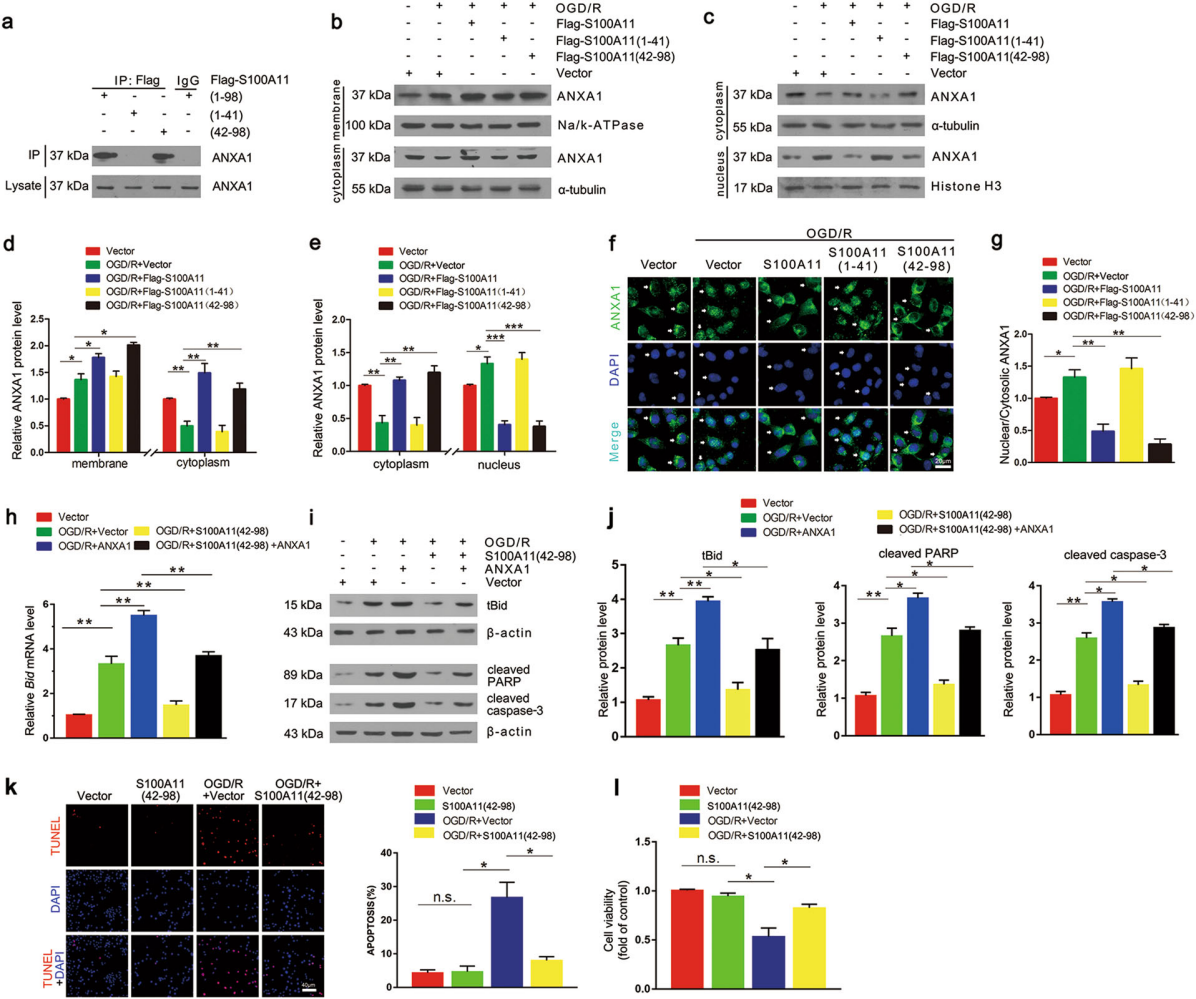
Amino acids 42–98 of S100A11 are required to specifically inhibit the nuclear translocation of ANXA1 and decrease the expression of apoptosis-related proteins. **a** Co-IP showing the interaction of ANXA1 with S100A11 in N2a cells transfected with Flag-S100A11 (WT), (1–41), and (42–98) plasmids. **b**–**e** Western blot showing the subcellular localization of ANXA1 in N2a cells transfected with Flag-S100A11 (WT), (1–41), and (42–98) plasmids after OGD/R, and the quantitative analysis of subcellular ANXA1 levels. Data are reported as the means ± S.E.M. from three independent experiments. **f**, **g** Images of immunofluorescence staining showing the subcellular localization of ANXA1 in N2a cells transfected with Flag-S100A11 (WT), (1–41), and (42–98) plasmids after OGD/R, and quantitative analysis of nuclear/cytoplasmic ANXA1 levels. Scale bar = 20 μm. **h** qPCR analysis of *Bid* mRNA expression in N2a cells transfected with Flag-S100A11 (42–98) and/or GFP-ANXA1 plasmids after OGD/R. **i**, **j** Western blots showing the levels of tBid, cleaved PARP and cleaved caspase-3 proteins in N2a cells and statistical analysis of apoptosis-related protein expression. **k**, **l** TUNEL staining (**k**) and MTT assay (**l**) showing the effect of S100A11 (42–98) on the apoptosis and viability of N2a cells subjected to OGD/R. n.s: the difference between the two groups was not significant; *P < 0.05; **P *<* 0.01; ***P *<* 0.001. Data are reported as the means ± S.E.M. from three independent experiments

Based on these findings, we further explored the role of S100A11 after OGD/R. As described in previous experiments, we generated different S100A11 mutants. Co-IP experiments revealed that Flag-S100A11-WT and (42–98), but not (1–41), bound to ANXA1 (Fig. 8a), implying that Flag-S100A11-(42–98) was required for S100A11 binding to ANXA1. To address the effect of S100A11 fragmentation on ANXA1 translocation, western blotting showed S100A11 overexpression promotes ANXA1 protein membrane and cytoplasmic accumulation, but only a small amount in the nuclear after OGD/R (Fig. 8b–e). However, S100A11-(1–41) did not influence ANXA1 subcellular levels under OGD/R conditions, whereas S100A11-(42–98) mimicked the biological function of full-length S100A11 after OGD/R. Immunofluorescence staining showed the Similar data (Fig. 8f, g). We further explored S100A11-(42–98) on cell apoptosis involved ANXA1 (Fig. 8h–j). The increased levels of the *Bid* mRNA and apoptosis-related proteins in the group transfected with ANXA1 were significantly reversed by transfection with S100A11-(42–98) after OGD/R. Next, TUNEL staining showed that S100A11-(42–98) overexpression obviously decreased the number of apoptotic cells following OGD/R (Fig. 8k). In addition, an MTT assay that OGD/R-induced decrease in cell viability was reversed by S100A11-(42–98) overexpression (Fig. 8l). Based on our results, S100A11-(42–98) protects against cell apoptosis by inhibiting the nuclear translocation of ANXA1 after OGD/R.

## Discussion

**Fig. 9:**
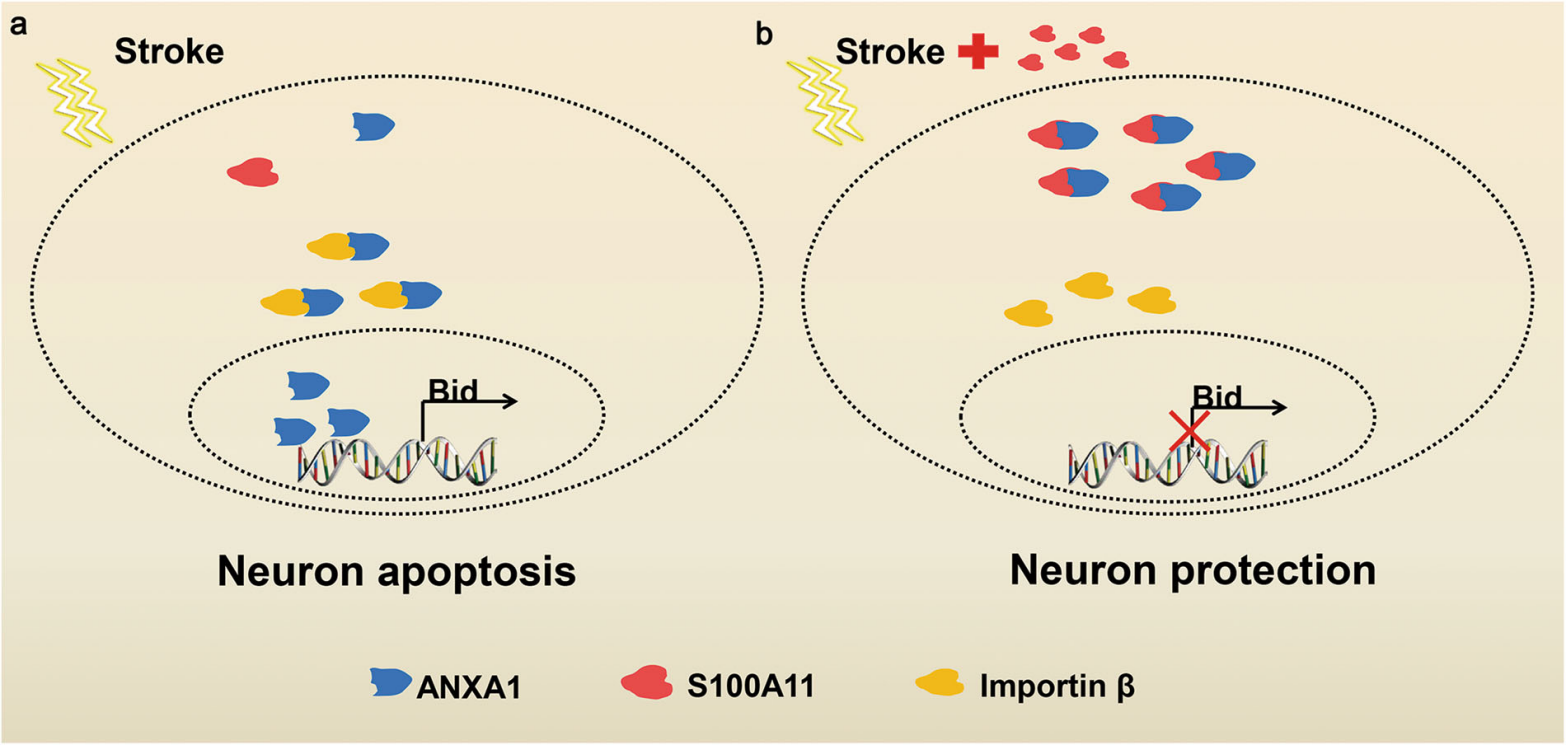
Schematic representation of the protective effects of S100A11 overexpression on neuronal cell apoptosis induced by ischemic stroke. **a** The stroke-induced decrease in S100A11 expression causes the nuclear accumulation of ANXA1, which ultimately activates the apoptosis pathway. **b** In comparison, overexpression of exogenous S100A11 promotes ANXA1 membrane translocation and inhibits ANXA1 nuclear translocation by competing with importin β for NTS sequence of ANXA1, ultimately protecting neuronal cells from stroke-induced apoptosis

Cerebral ischemia markedly increases morbidity and mortality^34,35^. Therefore, the discovery of novel drugs for the treatment of ischemic stroke is critically needed. Based on accumulating evidence, the nuclear translocation of ANXA1 induces cell apoptosis, which is an important neuropathological process leading to neuronal damage after cerebral ischemia^5,6^. This finding prompted us to search for new targets with neuroprotective effects on ANXA1-induced cell apoptosis after stroke. In this study, we elucidated the effects of S100A11 on protecting against neuronal apoptosis induced by ANXA1 nuclear translocation after ischemic stroke (Fig. 9a, b). Specifically, after OGD/R, S100A11 silencing results in ANXA1 transport from the cytoplasm to the nucleus, whereas S100A11 overexpression leads to ANXA1 membrane accumulation. As S100A11 is a typical cytoplasmic protein and an important adaptor protein for ANXA1, it promotes ANXA1 translocation to the membrane through a PEX14-dependent mechanism. Once S100A11 accumulated in the cytoplasm following PEX14 silencing, then S100A11 decreased nuclear accumulation of ANXA1 by inhibiting the ANXA1-importin β interaction through an interaction with the NTS sequence of ANXA1. Subsequently, nuclear translocation of ANXA1 decreased, and then *Bid* mRNA expression and the activity of the caspase-3 apoptosis pathway decreased. In particular, S100A11 overexpression improved cell survival following OGD/R. More importantly, an i.c.v. injection of Ad-S100A11 *in vivo* improved cognitive function of mice after MCAO. The stroke experiments were performed using the filament model of stroke in which ischemia-reperfusion injury might be much more prevalent than in human stroke. Therefore, whether the impressive efficacy of S100A11 overexpression would probably translate to human stroke still remained to be discussed in further research work.

In the present study, we sought to determine the effect of S100A11 on ANXA1 translocation after OGD/R as well as the associated mechanism. S100A11 overexpression reduced ANXA1 nuclear translocation by inhibiting the interaction of ANXA1 with importin β. First, accumulation of S100A11 in the cytoplasm upon silencing of PEX14, an essential molecule for S100A11 membrane translocation, primarily increased the level of cytoplasmic ANXA1 but did not alter the level of nuclear ANAX1. Thus, the nuclear translocation of ANXA1 was diminished in a S100A11-dependent manner. Furthermore, the truncated S100A11 fragment (S100A11-(42–98)), which contains the binding site for ANXA1, reduced ANXA1 nuclear migration. More importantly, N-terminal fragment of ANXA1, which is known to contain the binding sequence for S100A11, did not change the interaction between ANXA1 and S100A11. However, the ANXA1-S100A11 interaction was blocked when both the N-terminal fragment and the NTS sequence of ANXA1 were truncated, as the NTS sequence is essential for ANXA1 nuclear translocation^6^. Thus, S100A11 is associated with neuronal survival by regulating ANXA1 nuclear translocation.

Many factors are responsible for inducing membrane and nuclear translocation in response to a specific stimulus; for example, oxidative stress^36^ and PKC^37^ promote ANXA1 nuclear translocation. In comparison, lipopolysaccharides^38^, glucocorticoids^15^, and hepatocyte growth factors induce ANXA1 membrane translocation. ANXA1 nuclear translocation was recently shown to be involved in neuronal apoptosis^5,6,10^ and ANXA1 membrane translocation has been implicated in neuronal survival after OGD/R. According to the results from our current study, S100A11 overexpression promotes a direct interaction between S100A11 and ANXA1 after OGD/R. This direct interaction may largely reduce ANXA1 nuclear translocation by competing with importin β for the NTS sequence of ANXA1, but not the N-terminal fragment of ANXA1. Importantly, the N-terminal fragment of ANXA1 is not the sole binding site for S100A11^29,39^.

OGD/R-induced TRPM7 activation largely accelerates Ca^2+^ influx into the cytoplasm^40–42^, promoting the interaction of ANXA1 and S100A11^26,43^. Consistent with this possibility, we speculated that S100A11 overexpression would promote ANXA1 membrane and cytoplasmic accumulation after OGD/R. Second, S100A11 and ANXA1 proteins have recently been shown to protect against cell membrane damage and promote survival of cancer cells^27,44^. Interestingly, in the present study, S100A11 induced ANXA1 membrane accumulation. However, we have not determined whether ANXA1 located within the cell membrane is secreted into the extracellular space, and if so, whether it promotes the production of anti-inflammatory factors via the interaction with formyl peptide receptors on microglia, ultimately protecting against neuronal injury after OGD/R. Third, the current study was performed using neurons and N2a cells. Different cells may respond to the same treatment with different reactions. Therefore, their responses to OGD/R may be distinct from the response of immunocytes. Future studies in our laboratory will address these issues. To the best of our knowledge, this study is the first to show that S100A11 directly participates in and even inhibits the process of ANXA1 translocation to the nucleus and the first step to verify that S100A11 protects against ischemic stroke by regulating ANXA1 subcellular localization and provides a broader view of S100A11 function after MCAO. Based on our findings, treatments that increase the interaction between S100A11 with ANXA1 may represent a promising therapeutic strategy for cerebral ischemia.

## Materials and methods

### Animals

C57BL/6J wild-type male mice (25 ± 2 g) were used throughout the study and housed (five per cage) in a quiet room in the animal care facility. The feeding environment was maintained at 22 ± 2 °C on a 12-h light/dark cycle. During the experimental period, all the mice were provided water and standard animal food. All the procedures were approved by the IACUC committee of Huazhong University of Science and Technology.

### Cerebral ischemia mouse model

The mice underwent focal cerebral ischemia, which is a MCAO surgery. Mice were anesthetized by using 350 mg/kg of 4% chloral hydrate (i.p.). In brief, we surgically exposed the left external carotid artery (ECA), common carotid artery (CCA), and internal carotid artery (ICA) through a midline neck incision. The CCA was ligated distally, and the ECA was ligated at two locations, at the distal end of the ECA and near the bifurcation of the ICA and ECA, using surgical nylon monofilaments. A mini-incision was then made between the two ECA ligatures. A 2-cm length of nylon filament (diameter 0.25 ± 0.03 mm) with a red mark at 1 cm was gently inserted into the lumen of the ICA from the left ECA up to approximately the location of the mark. After 60 min, the filament was withdrawn, and amoxicillin was used to prevent bacterial infection. The sham groups underwent the same procedure without obstruction of the left CCA. The mice were returned to their cages after the procedure and sacrificed after 24 h.

### Ad-S100A11 intracerebroventricular injection

Adenoviruses encompassing the *Mus musculus* S100A11 gene (Ad-S100A11) (Vigenebio, Shandong, China) were injected intracerebroventricularly (i.c.v.) into the left hemisphere (anteroposterior, 0.23 mm; lateral, 1.0 mm; depth, 2.2 mm; from bregma) using stereotaxic methods 72 h before MCAO. The injections were conducted with a stepper-motorized microsyringe (Hamilton, Bonaduz, Switzerland) at a rate of 1 μl/min. Amoxicillin was used to prevent bacterial infection. The control mice received the same dose, but the adenoviruses were replaced with GFP.

### TUNEL assay

TUNEL staining was applied to examine cell apoptosis in 10-μm frozen brain sections according to the manufacturer’s instructions (Roche, Basel, Switzerland). Individual TUNEL^+^ cells were counted in consecutive 1 mm^2^ fields in the ischemic penumbra of six randomly selected mice from each group. The number of TUNEL^+^ cells per area in 20 successive fields of view in four sections from each mouse was counted by an observer who was blinded to the study design, and the mean number of TUNEL^+^ cells in the fields of view was calculated for each mouse.

### Flow cytometry

The proportion of apoptotic cells were evaluated using a FITC-Annexin V Apoptosis Detection Kit (BD Pharmingen, San Diego, CA, USA). In brief, neurons were harvested through trypsinization, washed two times with cold phosphate-buffered saline (PBS) containing 2% bovine serum albumin (BSA), resuspended in binding buffer at a density of 5 × 10^5^ cells/500 μl, and then incubated with 5 μl of FITC-conjugated Annexin V and 5 μl of propidium iodide for 15 min at room temperature in the dark. The samples were analyzed by flow cytometry using a BD FACSCalibur^TM^ flow cytometer (Becton Dickinson, San Jose, CA).

### N2a cell culture, transfection, and OGD/R model

Cells were grown in Dulbecco’s modified Eagle’s medium (DMEM) supplemented with penicillin, streptomycin, and 10% fetal bovine serum (FBS) (Gibco, Gaithersburg, MD, USA). The cultivation environment was maintained at 5% CO_2_ and 37 °C. Neuro-2a (N2a) cells were transfected with the indicated plasmids using Lipofectamine 2000 (Invitrogen, Carlsbad, CA, USA), according to the manufacturer’s instructions. After 24 h, N2a cells were exposed to OGD conditions. The cultivation media was substituted with glucose-free DMEM (Gibco, Gaithersburg, MD, USA), and cells were placed in an incubator with 5% CO_2_ and 95% N_2_ at 37 °C. After 60 min, the medium was replaced with maintenance medium and returned to an incubator containing 5% CO_2_ at 37 °C for 24 h.

### Primary cortical neuron culture

The protocol for the use of rats for neuronal cultures was performed according to the principles of the Animal Care Committee of Huazhong University of Science and Technology. In brief, cerebral cortices were isolated from mouse embryos aged 16–18 days under a dissection microscope, cut into ~ 1-mm^3^ pieces, and incubated with 0.25% trypsin-ethylenediaminetetraacetic acid for 15 min at 37 °C. Freshly prepared nutrient mixture F12 (DMEM-F12) medium containing 10% FBS (Gibco, Gaithersburg, MD, USA) was used to terminate the trypsin digestion. Subsequently, fire-polished glass pipettes were used to gently triturate the tissue mass into a cell suspension. Finally, cells were counted and plated on poly-L-lysine-coated six-well plates at a density of 1 × 10^7^cells per well. Neurons were cultured at 37 °C in a humidified atmosphere containing 5% CO_2_. 24 h later, the culture medium was replaced with Neurobasal medium supplemented with 2% B-27, and the culture media were refreshed two times per week. Neurons were used for the experiments between days 7 and 10 in vitro.

### Neurological deficits

Neurological tests were performed at 72 h after stroke by an observer who was blinded to the study design. The following neurological grading score was used: 0 = no deficit, 1 = torso flexion to the contralateral side, 2 = spontaneous circling to the contralateral side, 3 = falling to the contralateral side and 4 = no spontaneous movement.

### TTC staining

TTC staining was performed 24 h after MCAO surgery. The brains of mice in each group were rapidly extracted and cut into six 2-mm thick slices beginning at the frontal region. Subsequently, sections were placed in a 1% TTC (Sigma-Aldrich, St. Louis, MO, USA) solution for 30 min at 37 °C and then submerged in 4% paraformaldehyde for fixation. The dark red regions corresponding to normal tissue and white regions corresponding to infarcted areas were analyzed using ImageJ software (NIH, Baltimore, MD, USA). The infarcted areas of each section were summed and presented as a percentage of the volume of uninfarcted areas.

### Behavioral testing

#### Rotarod test

In brief, after the operation, mice were allowed to practice walking on a rotating rod at a speed of 10 revolutions per minute (rpm) for 3 days. On the fourth day, we evaluated the ability of the mice of each group to walk on the rotarod, which was accelerated from 4 to 40 rpm for 5 min. The time at which each mouse fell off the rod was recorded as the retention time. Each mouse underwent three trials, and the data were subjected to statistical analysis.

#### Open field test

Open field tests were conducted using a TM-Vision system. In brief, the system consists of a 50 × 50 × 30 cm^3^ black box, a dark surround and a computer-controlled camera. At the beginning of each trial, each animal was placed at the center of the device. The total distance traveled and the time spent in the center were recorded during the 10-min trial.

#### Morris water maze

Each of the experimental animals was brought to a behavior room, which was maintained at 25 °C (the housing area during the training), and allowed a 2-day acclimation period. The full training period lasted for 7 days. Water was colored by adding milk to make the water opaque to the C57BL/6J mice. In brief, the mice were given 60 s to find the platform and were allowed to remain on the platform for 15 s to facilitate learning and directional memory. On the first 6 days, four trials were conducted, and the mice were released from four different random release points for each trial. The time spent in the target quadrant and the number of times the mouse crossed the target platform location during the probe trials on the seventh day of training were recorded to reflect learning and memory. In this study, behavioral tests were conducted by an experimenter who was blinded to the experimental group of each mouse.

#### Western blotting

The total protein was harvested 24 h after OGD/R. The samples were separated on 12% polyacrylamide gels and transblotted onto polyvinylidene difluoride membranes (Millipore). Membranes were incubated with 5% BSA for 1 h at room temperature and then incubated with primary antibodies overnight at 4 °C. After a 1-h incubation with secondary antibodies, levels of target proteins were quantified using an enhanced chemiluminescence kit, according to the manufacturer’s instructions (Pierce, Rockford, IL, USA). The levels of target proteins were quantified using ImageJ software after standardizing the expression levels to the level of α-tubulin or β-actin. The following primary antibodies were used: anti-S100A11 (ab180593, 1:500, Abcam), anti-ANXA1 (sc-11387, 1:1000, Santa Cruz Biotechnology), anti-histone H3 (4499, 1:1000, Cell Signaling Technology), anti-cleaved caspase-3 (9664, 1:1000, Cell Signaling Technology), anti-importin β (51186, 1:1000, Cell Signaling Technology), anti-tBid (ab10640, 1:1000, Abcam), and anti-β-actin (sc-47778, 1:2000, Santa Cruz Biotechnology), anti-cleaved PARP (5625, 1:1000, Cell Signaling Technology), anti-α-tubulin (sc-53646, 1:2000, Santa Cruz Biotechnology), anti-GFP (sc-9996, 1:2000, Santa Cruz Biotechnology), anti-PEX14 (ab109999, 1:1000, Abcam), anti-Na-K/ATPase (3010, 1:1000, Cell Signaling Technology), anti-His (9991, 1:1000, Cell Signaling Technology), and anti-Flag (8146, 1:1000, Cell Signaling Technology).

#### Co-immunoprecipitation

Total proteins were collected from N2a cells as described above. Cell lysates were diluted fourfold with IP buffer (Beyotime Institute of Biotechnology) containing a protease inhibitor cocktail. Briefly, an aliquot of the cell lysate was incubated with an anti-Flag, anti-GFP, or anti-ANXA1 antibody overnight at 4 °C, followed by an incubation with Protein A/G plus agarose (Santa Cruz Biotechnology, USA) for 4 h at 4 °C. The samples were washed five times with wash buffer. Precipitates were then boiled with 1 × sodium dodecyl sulfate sample buffer for 5 min. Levels of each protein in the samples were analyzed by western blotting.

#### Generation of plasmids

The mouse full-length S100A11 coding sequence was cloned into pFlag-CMV2, the ANXA1 coding sequence was cloned into pEGFP-N1, and PEX14 coding sequence was cloned into pcDNA3.1^+^ using recombinase connection method. Subsequent mutants were generated by recombination using Trelief SoSoo Cloning Kit Ver.2 according to the instructions of the manufacturer (TSINGKE, Beijing, China). A list of primers can be found in supplementary Table 1. In brief, subsequent mutants (ANXA1-ΔN, amino acids 1–26 deleted; ANXA1-ΔC, amino acids 27–346 deleted; ANXA1-ΔNTS, amino acids 228–237 deleted; ANXA1-ΔN-ΔNTS, amino acids 1–26 and 228–237 deleted; S100A11 (1–41), amino acids 42–98 deleted) were generated. Mouse ANXA1 (GenBank No. NM_000700.2) shRNA plasmids were purchased from GenePharma (Suzhou, Wuhan, China). shANXA1#1 was 5′-AGCGCCAGCAGATCAAGGC-3′ and shANXA1 #2 was 5′-GCCTCACAACCATCGTGAAGT-3′; shS100A11#1 was 5′-CTGAATTCCTTTCCTTCAT-3′ and shS100A11 #2 was 5′-GTGTCCTTGACCGCATGAT-3′.

#### Fluorescence resonance energy transfer assay

Restriction Enzymes of *Xho*1 and *Not*1 were used to digest pEGFP-N1-ANXA1 and its mutants in order to cleavage GFP. Next, sequences for full-length of CFP: Cyan Fluorescent Protein were amplified by PCR from pECFP-N1 and inserted in pEGFP-N1-ANXA1 and its mutants instead of GFP, generating pANXA1-CFP, pANXA1-ΔN-CFP, pANXA1-ΔNTS-CFP, and pANXA1-ΔN-ΔNTS-CFP, respectively. Sequence for S100A11 was amplified by PCR from pFlag-S100A11 and inserted upstream and in frame with the yellow fluorescent protein (YFP) in pEYFPN1, generating pS100A11-YFP. Plasmids pANXA1-CFP, pANXA1-ΔN-CFP, pANXA1-ΔNTS-CFP, or pANXA1-ΔN-ΔNTS-CFP were co-transfected with pS100A11-YFP into N2a cells, respectively. After 24 h transfection, cells were washed twice with PBS and fixed using 4% paraformaldehyde in PBS. Photobleaching was performed by repeated scanning a region of interest (ROI) using the 514 nm at maximum intensity. An excitation wavelength of 458 nm and an emission wavelength of 470–500 nm were used for CFP, and an excitation wavelength of 514 nm and an emission wavelength of 515–545 nm were used for YFP. The FRET energy transfer efficiency (Ef) was calculated as FRET_eff_ = (I_post_−I_pre_)/I_post_ where I_pre_ and I_post_ are the total fluorescence of the ROI before and after bleaching. All images were collected with a Zeiss double-photon fluorescence microscope (Zeiss 510 Meta, Oberkochen, Germany).

### Statistical analysis

Data are expressed as means ± S.E.M. from at least three independent experiments. Two-way analysis of variance was initially performed to determine any differences among treatments, and non-parametric *t* tests were used for comparisons of two groups. *P* values < 0.05 were considered statistically significant.

## Electronic supplementary material

Supplementary Information

Supplementary Figure 1

Supplementary Figure 2

Supplementary Figure 3

Supplementary Figure 4
