# Supplementary material for: S100A11 protects against neuronal cell apoptosis induced by cerebral ischemia via inhibiting the nuclear translocation of annexin A1
Source: Cell Death Dis. 2018 May 29;9(6):657. doi: 10.1038/s41419-018-0686-7 (PMC5974363; doi:10.1038/s41419-018-0686-7)
Supplement: Supplementary file 1 — Supplementary Information [file 41419_2018_686_MOESM1_ESM.doc]

Supplementary Information for:

**S100A11 protects against neuronal cell apoptosis induced by cerebral ischemia via inhibiting the nuclear translocation of annexin A1**

**Qian Xia^1,2,3,4^, Xing Li^1,2,3,4^, Huijuan Zhou^1,2,3^, Lu Zheng^1,2,3^, Jing Shi^*,1,2,3^**

**Supplementary figure legends:**

Supplementary Figure 1: Adenoviral transfection of S100A11 enhances ANXA1 expression. (**a** and **b**) C57 mice were administered an intracerebroventricular injection of either Ad-GFP or Ad-S100A11 adenoviral particles for 72 h. Western blotting assay (**a**) and immunofluorescence analysis (**b**) were conducted to test the transfection efficiency, and the data were statistically analyzed. ^**^*P* < 0.01 versus control. Data are reported as the mean ± S.E.M. from three independent experiments.

Supplementary Figure 2: The interference efficiency of shS100A11 and shANXA1. (**a**) Western blotting showing the interference efficiency of shS100A11. (**b**) Western blotting showing the interference efficiency of shANXA1. ^*^*P* < 0.05 and ^**^*P* < 0.01 versus control. Data are reported as the mean ± S.E.M. from three independent experiments.

Supplementary Figure 3: The interference efficiency of shPEX14 and the overexpression level of His-PEX14. (**a**) Effect of shPEX14 on *PEX* mRNA expression. RT-PCR showing the mRNA level of PEX14 in N2a cells pretreated with shPEX14 plasmids and the quantitative analysis of mRNA levels of S100A11. (**b**) Western blotting showing that the expression level of PEX14 in N2a cells transfected with His-PEX14 plasmid. ^**^*P* < 0.01 versus control group. Data are reported as the mean ± S.E.M. from three independent experiments.

Supplementary Figure 4: The protein-protein interactions of BIFC constructs in N2a cells. Cells were co-transfected with blank Venus constructs-VN and VC or S100A11-VN and VC or VN and ANXA1-VC, only a few cells fluoresced in cells (A, B, C). In contrast, cells co-transfected with S100A11-VN and ANXA1-VC or ANXA1-ΔN-VC or ANXA1-ΔNTS-VC (D, E, F) fluoresced in many cells. But cells co-transfected with S100A11-VN and ANXA1-ΔN-ΔNTS-VC, a few cells fluoresced in cells (G). Venus signals were analyzed.

Supplementary Table 1: The primers were used for construction of pEGFP-ANXA1 and its mutants, pFlag-S100A11 and its mutants, pcDNA3.1-ANXA1-VC

and its mutants, pANXA1-CFP and its mutants, pcDNA3.1-His-PEX14and pcDNA3.1-S100A11-VN plasmids.

| Primer name | Sequence | Final Construct Name | Restriction endonuclease |
| --- | --- | --- | --- |
| ANXA1-FL-F  ANXA1-FL-R | 5’-agtgaaccgtcagatccgctagcatggcaatggtatca-3’ | pEGFP-ANXA1 | Nhe 1 and Xho 1  (Double Digests) |
|  | 5’-gcagaattcgaagcttgagctcgaggtttccaccacacag-3’ |  |  |
| ANXA1-delN26-F  ANXA1-delN26-R | 5’-agtgaaccgtcagatccgctagctcatacaa aggtggtc-3’ | pEGFP-ANXA1-ΔN | Nhe 1 and Xho 1  (Double Digests) |
|  | 5’-gcagaattcgaagcttgagctcgaggtttccaccacacag-3’ |  |  |
| ANXA1-delC(27-346)-F  ANXA1-delC(27-346)-R | 5’-agtgaaccgtcagatccgctagcatggcaatggtatca-3’ | pEGFP-ANXA1-ΔC | Nhe 1 and Xho 1  (Double Digests) |
|  | 5’-gcagaattcgaagcttgagctcgagttttacagcttgaac-3’ |  |  |
| ANXA1-ΔNTS-F1  ANXA1-ΔNTS-R1  ANXA1-ΔNTS-F2  ANXA1-ΔNTS-R2 | 5’-agtgaaccgtcagatccgctagcatggcaatggtatca-3’ | pEGFP-ANXA1-ΔNTS | Nhe 1 and Xho 1  (Double Digests) |
|  | 5’-gcagaattcgaagcttgagctcgaggctggtcagaattgtgg-3’ |  |  |
|  | 5’-tcttcaccacaattctgaccagcagaattacggaaagtacagtcaa-3’ |  |  |
|  | 5’-ttgactgtactttccgtaattctgctggtcagaattgtggtgaaga -3’ |  |  |
| ANXA1-ΔN26-ΔNTS-F1  A ANXA1-ΔN26-ΔNTS-R1  ANXA1-ΔN26-ΔNTS-F2  ANXA1-ΔN26-ΔNTS-R2 | 5’-agtgaaccgtcagatccgctagctcatacaa aggtggtc-3’ | pEGFP-ANXA1-ΔN-ΔNTS | Nhe 1 and Xho 1  (Double Digests) |
|  | 5’-gcagaattcgaagcttgagctcgaggctggtcagaattgtgg-3’ |  |  |
|  | 5’-tcttcaccacaattctgaccagcagaattacggaaagtacagtcaa-3’ |  |  |
|  | 5’-ttgactgtactttccgtaattctgctggtcagaattgtggtgaaga -3’ |  |  |
| S100A11- delN41-F  S100A11- delN41-R | 5’- cgatgacgacaagcttgcggccgcatgcctacagagactg-3’ | pFlag-S100A11-C(42-98) | Not1 and BamH1  (Double Digests) |
|  | 5’-agggatgccacccgggatccgattcgcttctgggaa-3’ |  |  |
| S100A11- delC(42-98)-F  S100A11- delC(42-98)-R | 5’-cgatgacgacaagcttgcggccgcatgcctacagagactg-3’ | pFlag-S100A11-N(1-41) | Not1 and BamH1  (Double Digests) |
|  | 5’-agggatgccacccgggatccctctgtttcatgaag-3’ |  |  |
| S100A11-FL-F  S100A11-FL-R | 5’-cgatgacgacaagcttgcggccgcatgcctacagagactg-3’ | pFlag-S100A11 | Not1 and BamH1  (Double Digests) |
|  | 5’-agggatgccacccgggatccgattcgcttctgggaa-3’ |  |  |
| PEX14-FL-F  PEX14-FL-R | 5’-agggagacccaagctggctagcatggcgtcgtcggagc-3’ | pcDNA3.1-His-PEX14 | Nhe 1 and Xho 1  (Double Digests) |
|  | 5’-ttaaacgggccctctagactcgaggtctcgctcagtctca-3’ |  |  |
| S100A11-FL-F  S100A11-FL-R | 5’-gccgccactgtgctggatatcatgcctacagagactg-3’ | pcDNA3.1-S100A11-VN | EcoR V  (single restriction digest) |
|  | 5’-ccagtgtggaattctgcagatatcgattcgcttctgggaa-3’ |  |  |
| ANXA1-FL-F  ANXA1-FL-R | 5’-gccgccactgtgctggatatcatggcaatggtatcagaatt-3’ | pcDNA3.1-ANXA1-VC | EcoR V  (single restriction digest) |
|  | 5’-ccagtgtggaattctgcagatatcgtttccaccacacag-3’ |  |  |
| ANXA1-delN26-F  ANXA1-delN26-R | 5’-gccgccatcgtgctggatatctcatacaaaggtggtc-3’ | pcDNA3.1-ANXA1-ΔN-VC | EcoR V  (single restriction digest) |
|  | 5’-ccagtgtggaattctgcagatatcgtttccaccacacag-3’ |  |  |
| ANXA1-delNTS-F  ANXA1-delNTS-R | 5’-gccgccatcgtgctggatatcatggcaatggtatcagaatt-3’ | pcDNA3.1-ANXA1-ΔNTS-VC | EcoR V  (single restriction digest) |
|  | 5’-ccagtgtggaattctgcagatatcgtttccaccacacag-3’ |  |  |
| ANXA1-delN26-delNTS-F  A ANXA1-delN26-delNTS-R | 5’-gccgccatcgtgctggatatcatggcaatggtatca-3’ | pcDNA3.1-ANXA1-ΔN-ΔNTS-VC | EcoR V  (single restriction digest) |
|  | 5’-ccagtgtggaattctgcagatatcgtttccaccacacag-3’ |  |  |
| ANXA1-FL-F  ANXA1-FL-R | 5’-gctctgtgtggtggaaacctcgagatggtgagcaagggc-3’ | pANXA1-CFP | Xho1 and Not 1  (Double Digests) |
|  | 5’-ctgattatgatctagagtcgcggccgcttacttgtacagctcgtcca-3’ |  |  |
| ANXA1-delN26-F  ANXA1-delN26-R | 5’-gctctgtgtggtggaaacctcgagatggtgagcaagggc-3’ | pANXA1-ΔN -CFP | Xho1 and Not 1  (Double Digests) |
|  | 5’-ctgattatgatctagagtcgcggccgcttacttgtacagctcgtcca-3’ |  |  |
| ANXA1-delNTS-F  ANXA1-delNTS-R | 5’-gctctgtgtggtggaaacctcgagatggtgagcaagggc-3’ | pANXA1-ΔNTS -CFP | Xho1 and Not 1  (Double Digests) |
|  | 5’-ctgattatgatctagagtcgcggccgcttacttgtacagctcgtcca-3’ |  |  |
| ANXA1-delN26-delNTS-F  ANXA1-delN26-delNTS-R | 5’-gctctgtgtggtggaaacctcgagatggtgagcaagggc-3’ | pANXA1-ΔN -ΔNTS -CFP | Xho1 and Not 1  (Double Digests) |
|  | 5’-ctgattatgatctagagtcgcggccgcttacttgtacagctcgtcca-3’ |  |  |

**Materials and Methods**

**Total protein extraction**

N2a whole cell lysates were extracted as previously described[^1^](#_ENREF_1). In brief, cells were immersed in radio immunoprecipitation assay (RIPA) lysis buffer for 15 minutes, and the entire process was performed on ice. The cell lysate was centrifugalized at 12 000 rpm for 15 minutes, and then the supernatant removed to obtain the product.

**Cell fractionation**

Separate membrane, cytoplasmic was prepared using a Membrane and Cytosol Protein Extraction Kit (Cat. P0033, Beyotime Institute of Biotechnology, Haimen, China). Brifly, N2a cells were Washed once with PBS, and then scrape cells with cell scrapes or treat cells with digestion solution containing EDTA but no pancreatin to keep cells from adhering and using a pipette to blow the cells down. Cells were centrifugated at 1 000 r.p.m for 5 min and then aspirate the supernatant. Cells precipitation was resuspended with cold PBS, and take a small amount of cells for counting, and residual cells were centrifugated with 600 g for 5 minutes. The supernatant was discarded, and Membrane Protein Extraction Reagent A was added into 20 million to 50 million cells and suspended gently, and leave it on the ice 15 minutes. Next, the cell suspension was transferred to a suitably sized precooled glass homogenizer, homogenized about 30-50 times until at least 70% of the cells have been broken, cell suspension was centrifugated at 700 g, 4 °C for 10 min and then carefully collect the supernatant into a new centrifuge tube. Cell suspension was centrifugated at 14 000 g, 4 °C for 30 min, and the supernatant was cytoplasmic protein. 200 μl of Membrane Protein Extraction Reagent B was added into the precipitate and votex for 5 s to suspend it，and centrifugated at 14 000 g, 4 °C for 5 min，and the supernatant was cell membrane protein.

Cell nuclear and cytoplasmic protein was extracted according to a Nuclear and Cytoplasmic Protein Extraction Kit (Cat. P0028, Beyotime Institute of Biotechnology). Brifly, N2a cells were Washed once with PBS, and then scrape cells with cell scrapes or treat cells with digestion solution containing EDTA but no pancreatin to keep cells from adhering and using a pipette to blow the cells down. Cells were centrifugated at 1 000 r.p.m for 5 min and then aspirate the supernatant. Cytoplasmic protein extraction reagent A was added into the precipitate for 200 μl per 20 μl cell pellet and vortex for 5 s fastly and leave it on ice for 15 min. Then add 10 μl of Cytoplasmic Protein Extraction Reagent B into the suspend and vortex for 5 s fastly and leave it on ice for 1 min, vortex for 5 s fastly. The suspend was centrifugated at 12 000 g, 4 °C for 5 min, the suspend was cytoplasmic protein. Next 50 μl of nuclear protein extraction reagent was added into the precipitate, vortex for 15 s fastly and leave it on ice for 2 min and then vortex for 15 s fastly, leave it on ice for 2 min, the operation was repeated for 30 min. And the suspend was centrifugated at 12 000 g, 4 °C for 10 min and the suspend was nuclear protein.

**Quantitative real-time PCR**

total RNA was extracted from preateated N2a Cells with the TRIzol reagent (Invitrogen), and cDNA was synthesized from 1 μg RNA using the ReverTra Ace-α-TM First Strand cDNA Synthesis Kit (Toyobo, Osaka, Japan). qPCR was performed with SYBR Green Real-Time PCR Master Mix (Toyobo) on a C1000 Thermal Cycler (Bio-Rad Laboratories, Hercules, CA, USA), according to the manufacturer’s recommendations. The primers used were as follows: Bid, 5′-GCCTGTCGGAGGAAGACAAA-3′ (forward) and 5′-GTGGAAGACATCACGGAGCA-3′ (reverse); β-actin, 5′-GGCTGTATTCCCCTCCATCG-3′ (forward) and 5′-CCAGTTGGTAACAATGCCATGT-3′ (reverse).

**Immunofluorescence staining**

The cultured N2a cells fixed in 4 % paraformaldehyde were thoroughly rinsed with PBS three times and treated with 10 % Triton X-100 for 10 min to rupture the cell membranes. The fixed cells were then blocked with 10 % donkey serum. One hour later, the coverslips were incubated overnight at 4 °C with the following primary antibodies: anti-NeuN (MAB377, 1:200, Millipore Biotechnology), anti-S100A11 (ab180593, 1:200, Abcam), and anti-annexin A1 (sc-11387, 1:200, Santa Cruz). After washing three times, the fixed coverslips were incubated with Alexa Fluor 488-conjugated AffiniPure goat anti-mouse IgG (H+L), Alexa Fluor 594-conjugated AffiniPure goat anti-mouse IgG (H+L) and Alexa Fluor 594-conjugated AffiniPure goat anti-rabbit IgG (H+L) (Jackson Immuno Research, West Grove, Pennsylvania, USA) for 60 min at room temperature. Finally, the fluorescence was captured by fluorescence microscopy (IX73, Olympus, Tokyo, Japan). Regional fluorescence intensities were quantified using Image Pro Plus software. To measure regional intensities, small circles within the cytoplasmic or nuclear regions of each cell were selected using the elliptical marquee tool. The intensity within each circle was obtained using the histogram function for each color channel, which was selected using the layers/channels palette. The values were recorded, for evaluation of the ratio of the nuclear to cytoplasmic intensity; we examined up to 10 independent fields of cells and scored at least 100 cells for each measurement.

**Bimolecular Fluorescence Complementation assay (BiFC)**

Cloning vectors pcDNA3.1-VN (1-157) and pcDNA3.1-VC(158-239), which express His-tagged-N-terminal 1-157 aa of Venus (VN) and His-tagged-C-terminal 158–239 aa of Venus (VC) fusion proteins, respectively, were kindly provided by Professor Xiao-Qian Chen (Huazhong University of Science and Technology, China). The mouse full-length S100A11 coding sequence was fused to the pcDNA3.1-VN (1-157) vector, (S100A11-VN); the full length ANXA1 coding sequence and its mutants were amplified by PCR from pEGFP-ANXA1, pEGFP-ANXA1-ΔN, pEGFP-ANXA1-ΔNTS and pEGFP-ANXA1-ΔN-ΔNTS and inserted in pcDNA3.1--VC(158-239), generating pcDNA3.1-ANXA1, pcDNA3.1-ANXA1-ΔN, pcDNA3.1-ANXA1-ΔNTS and pcDNA3.1-ANXA1-ΔN-ΔNTS, respectively. Subsequent mutants were generated by recombination using Trelief SoSoo Cloning Kit Ver.2 according to the instructions of the manufacturer (TSINGKE, Beijing, China). A list of primers can be found in supplemental Table 1. Fluorescence resulting from the interaction of the VN and VC proteins was detected by excitation at 515 nm and emission at 528 nm.

**Adenoviruses Vector Production**

All of the recombinant adenovirus vectors were based on type 5 (E1/E3 deficient) adenovirus. All of the Adenoviruses (Ad-S100A11, Ad-ANXA1) were purchased from Vigene (shangdong, China). The complete coding sequence of the S100A11 or ANXA1 were subcloned into the AsisI and MluI sites of the pENTER-CMV vector. The expression cassette of S100A11or ANXA1 were then transferred into the adenoviral backbone vector pAd, and the recombinant clones were confirmed by DNA sequencing. The recombinant viral vector of S100A11 or ANXA1 (Ad5-S100A11 and Ad5-ANXA1) was linearized by PacI digestion and packaged into HEK293 cells. Viral particles were purified by caesium chloride density gradient centrifugation and titred by TCID50 method. Ad5-EGFP was used to monitor the efficiency of cell infection and those with higher than 70% infection efficiency were used for further experiments.

**References**

1 Li, X. et al. Nuclear translocation of annexin 1 following oxygen-glucose deprivation-reperfusion induces apoptosis by regulating Bid expression via p53 binding. *Cell death & disease* **7**, e2356 (2016).
