# Supplementary figures and images for: S100A11 protects against neuronal cell apoptosis induced by cerebral ischemia via inhibiting the nuclear translocation of annexin A1
Source: Cell Death Dis. 2018 May 29;9(6):657. doi: 10.1038/s41419-018-0686-7 (PMC5974363; doi:10.1038/s41419-018-0686-7)

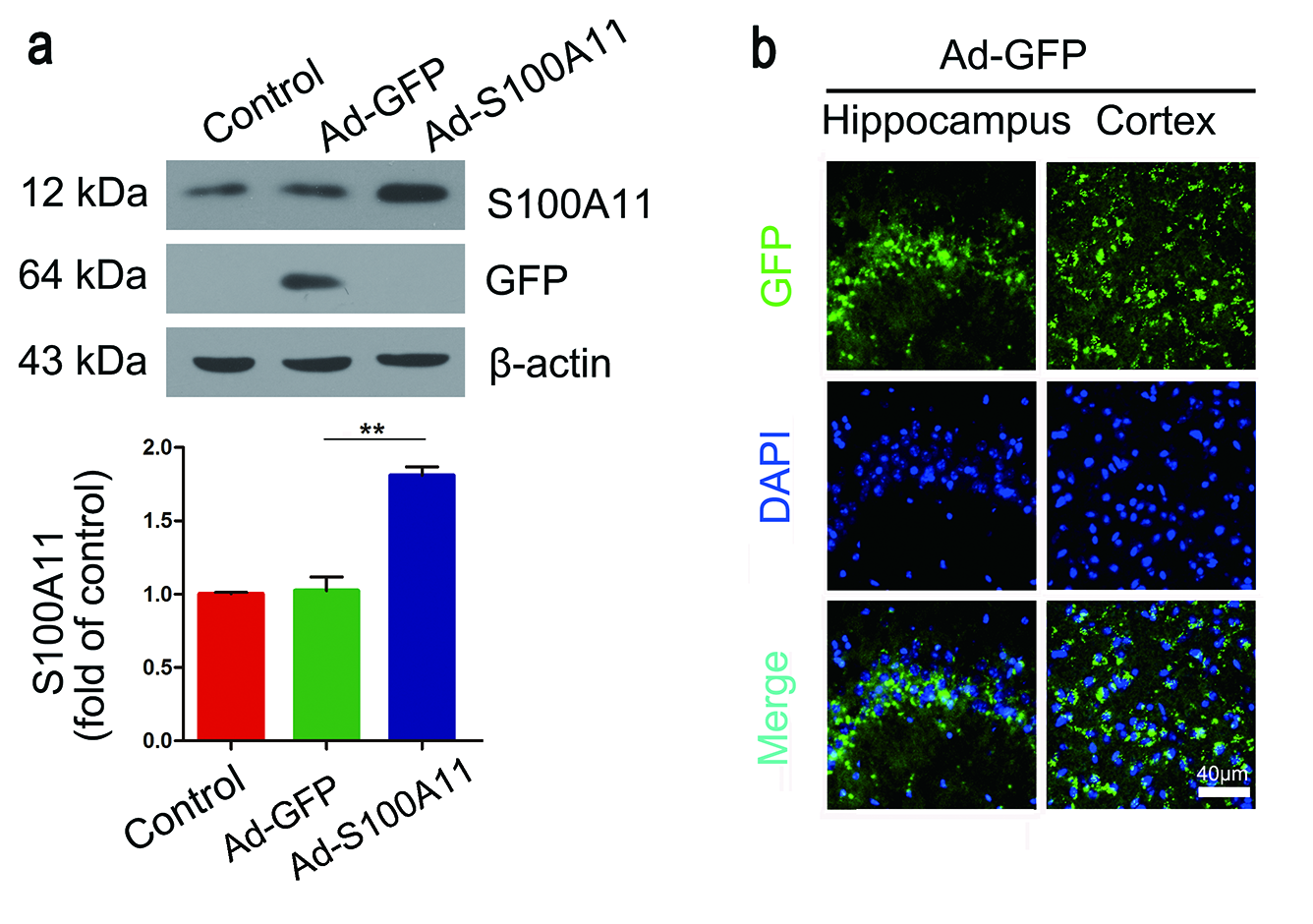

Supplement: Supplementary file 2 — Supplementary Figure 1 [file 41419_2018_686_MOESM2_ESM.tif]

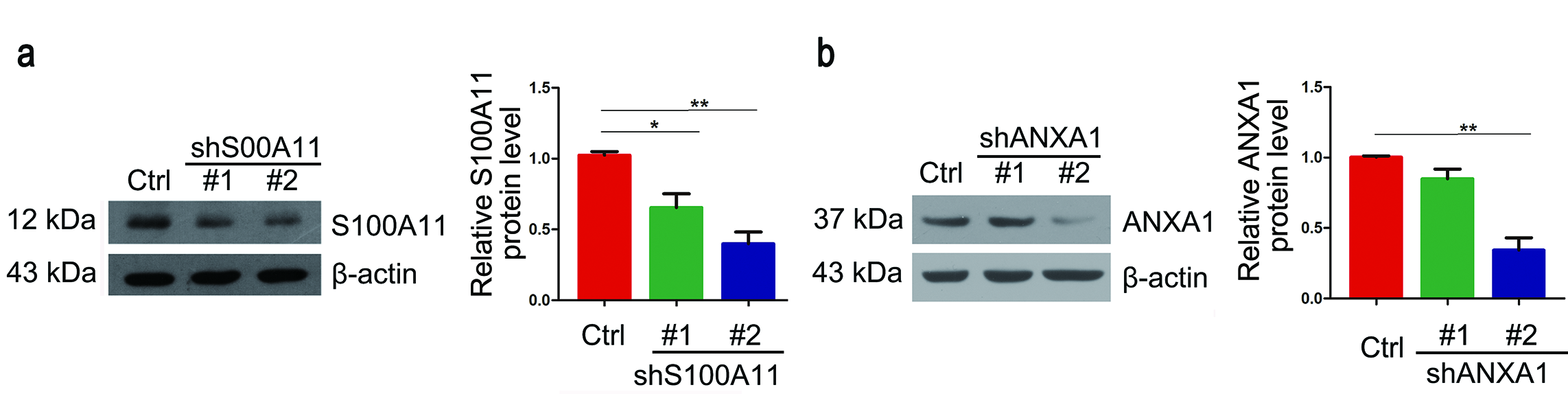

Supplement: Supplementary file 3 — Supplementary Figure 2 [file 41419_2018_686_MOESM3_ESM.tif]

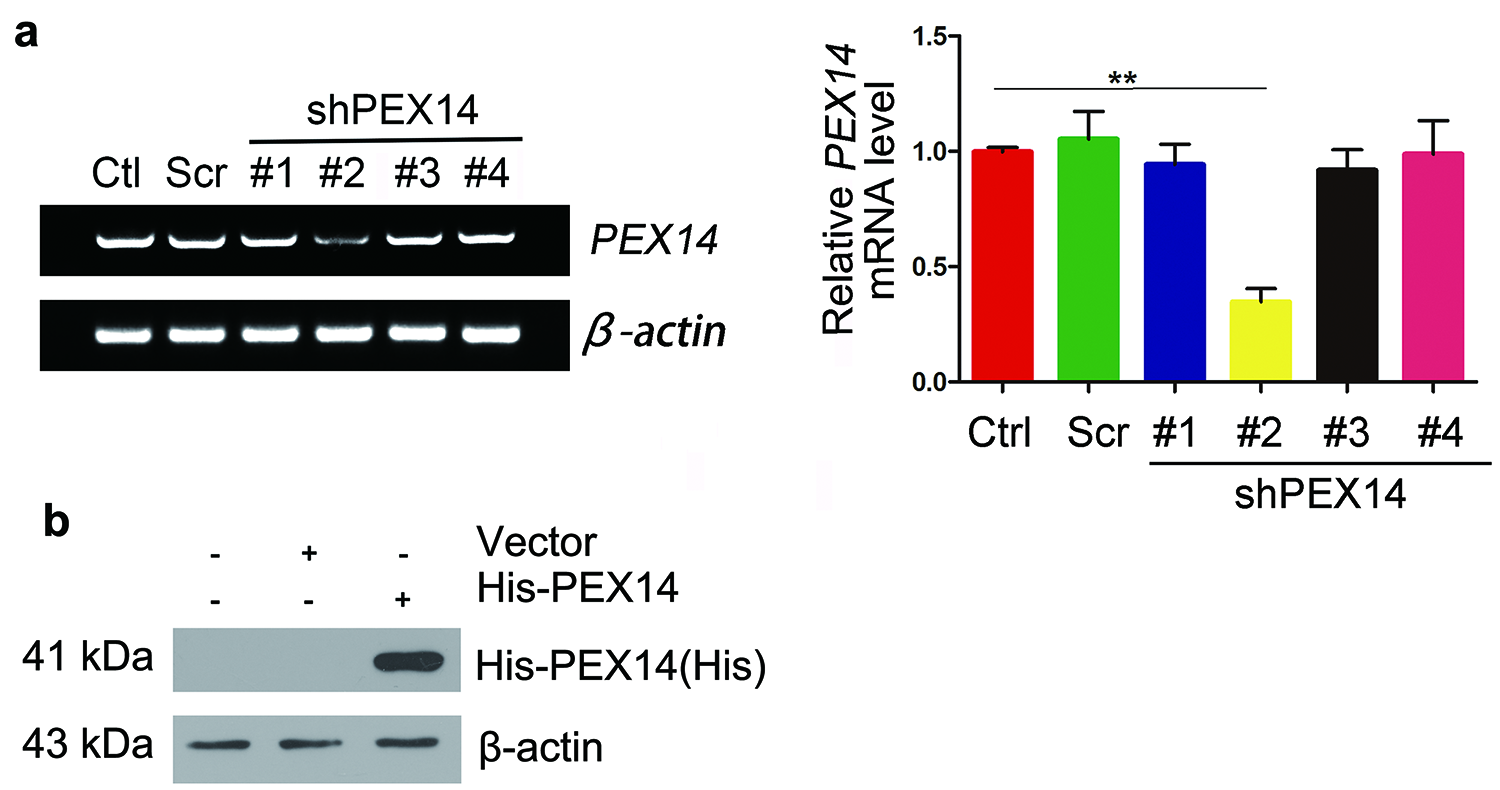

Supplement: Supplementary file 4 — Supplementary Figure 3 [file 41419_2018_686_MOESM4_ESM.tif]

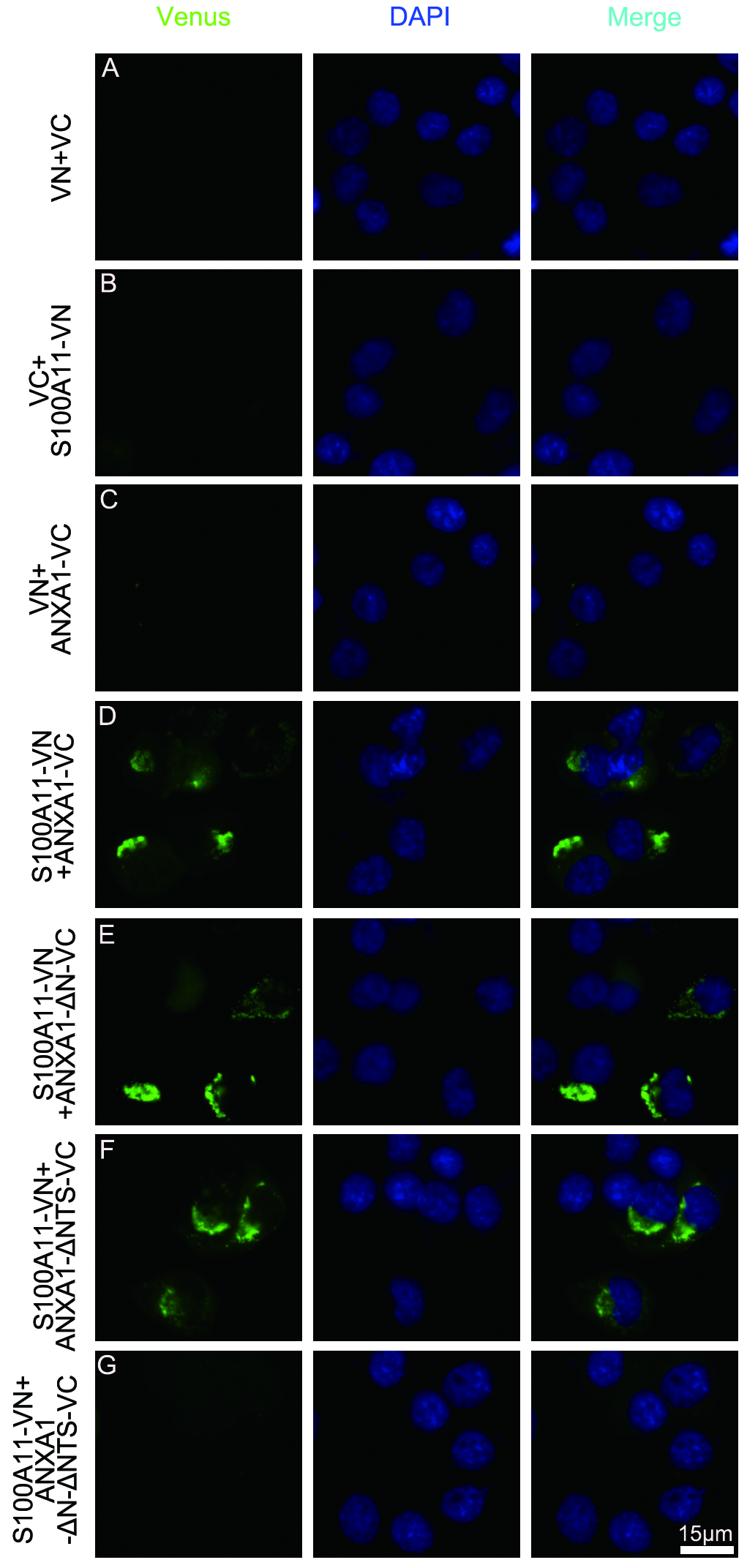

Supplement: Supplementary file 5 — Supplementary Figure 4 [file 41419_2018_686_MOESM5_ESM.tif]
